# Mini-G proteins: Novel tools for studying GPCRs in their active conformation

**DOI:** 10.1371/journal.pone.0175642

**Published:** 2017-04-20

**Authors:** Rony Nehmé, Byron Carpenter, Ankita Singhal, Annette Strege, Patricia C. Edwards, Courtney F. White, Haijuan Du, Reinhard Grisshammer, Christopher G. Tate

**Affiliations:** 1 MRC Laboratory of Molecular Biology, Cambridge, United Kingdom; 2 Membrane Protein Structure Function Unit, National Institute of Neurological Disorders and Stroke, National Institutes of Health, Department of Health and Human Services, Rockville, United States of America; Indian Institute of Technology Kanpur, INDIA

## Abstract

Mini-G proteins are the engineered GTPase domains of Gα subunits. They couple to GPCRs and recapitulate the increase in agonist affinity observed upon coupling of a native heterotrimeric G protein. Given the small size and stability of mini-G proteins, and their ease of expression and purification, they are ideal for biophysical studies of GPCRs in their fully active state. The first mini-G protein developed was mini-G_s_. Here we extend the family of mini-G proteins to include mini-G_olf_, mini-G_i1_, mini-G_o1_ and the chimeras mini-G_s/q_ and mini-G_s/i_. The mini-G proteins were shown to couple to relevant GPCRs and to form stable complexes with purified receptors that could be purified by size exclusion chromatography. Agonist-bound GPCRs coupled to a mini-G protein showed higher thermal stability compared to the agonist-bound receptor alone. Fusion of GFP at the N-terminus of mini-G proteins allowed receptor coupling to be monitored by fluorescence-detection size exclusion chromatography (FSEC) and, in a separate assay, the affinity of mini-G protein binding to detergent-solubilised receptors was determined. This work provides the foundation for the development of any mini-G protein and, ultimately, for the structure determination of GPCRs in a fully active state.

## Introduction

G protein-coupled receptors (GPCRs) are integral membrane proteins that play a ubiquitous role in intercellular communication throughout the human body. Binding of an agonist at the extracellular surface of the inactive receptor results ultimately in a conformational change at its intracellular surface that allows G protein coupling [1]. The conformation of the bound G protein is altered around the nucleotide binding pocket, resulting in GDP release, binding of GTP and subsequent activation of the G protein [2]. There has been great progress in the structure determination of GPCRs in the inactive state [3] bound to antagonists which highlights common structural features of the GPCR superfamily [4], a common mode of activation [5] and a conserved mechanism for the activation of G proteins [6]. However, it is still a major challenge to determine the structures of receptors in their fully active state, which can be defined structurally as the conformation bound to a heterotrimeric G protein or pharmacologically as the high-affinity agonist binding state of the receptor. The range of GPCR structures now available allows a clear distinction between agonist-bound structures either in the inactive state [7–10], in an active-intermediate state [11–14] or in an active state [2, 15–20]. It is, however, difficult to determine structures of receptors in an active state due to the instability of activated GPCRs and receptor-bound G proteins.

Four solutions have been developed to determine the active structures of GPCRs. The first active structure determined was of opsin stabilized at low pH [21] and bound to a C-terminal fragment of the G protein transducin [22]. This methodology has not been successful for any other GPCR. The second active state structure of a GPCR was determined of the β_2_-adrenergic receptor (β_2_AR) bound to a single-chain camelid antibody (nanobody) that mimics the pharmacological effects of a heterotrimeric G protein [15]. This has been applied successfully to other GPCRs [18, 19], but currently the methodology still requires the immunization of llamas with purified receptors [23], which makes it difficult to apply to many unstable GPCRs. The third methodology was to crystallise β_2_AR with the entire heterotrimeric G protein [2]. This was a great achievement and showed for the first time how a GPCR facilitated nucleotide exchange in the G protein. However, no other structures of a GPCR coupled to a heterotrimeric G protein have been published and the resolution of the GPCR portion of the structure was poor and required the previously determined active state structure of β_2_AR bound to a nanobody to allow the model to be built [2]. The fourth methodology was based on the observation that only the GTPase domain of the Gα subunit made significant contacts with active β_2_AR and therefore this domain was engineered and thermostabilised to generate a mini-G protein, mini-G_s_ [24]. Like the nanobodies, mini-G_s_ recapitulated all the receptor pharmacology upon coupling, but in addition, mini-G_s_ could couple to any G_s_-coupled receptor whereas nanobodies bound to only a specific receptor. The structure of the adenosine A_2A_ receptor (A_2A_R) coupled to mini-G_s_ was determined recently [20], which showed similar structural re-arrangements observed in the β_2_AR–G_s_ complex.

**Fig 1:**
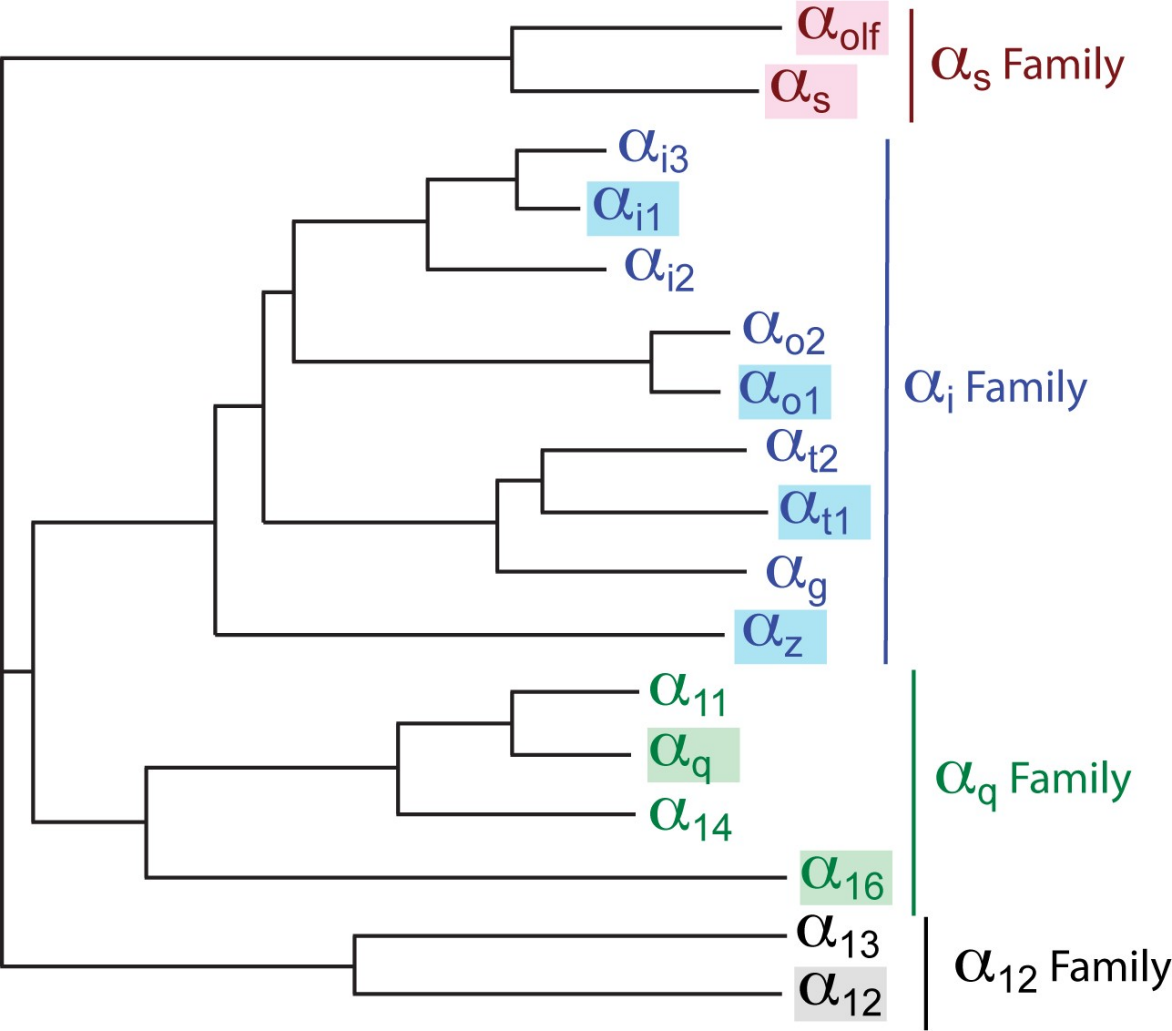
Phylogenetic relationship of human Gα subunits. All the Gα subunits that have been highlighted in the family-specific colours were attempted to be converted into mini-G proteins. The phylogenetic relationships were determined using TreeDyn.

The concept of mini-G proteins shows great promise for accelerating the rate of structure determination of GPCRs in their active states, from providing biophysical and pharmacological data on receptors in the active state, as well as providing a potential binding partner in co-crystallisation applications. However, there are four families of Gα subunits (Fig 1; Gα_s_, Gα_i_, Gα_q_, and Gα_12_) that show different specificities for various GPCRs [25]. Thus to be truly useful as tools in structural biology, at least one member from each family needs to be converted into a mini-G protein. Here we report the development of mini-G proteins for all the major Gα families. We also describe five different assays that can be used to characterize the binding of the mini-G proteins to GPCRs and show in three cases that the complexes can be purified by size exclusion chromatography. The two different methodologies for generating the mini-G proteins can be applied easily to any other Gα subunit, opening the doorway to studies on potentially any GPCR from any species.

## Materials and methods

### Ligands

The β_1_-adrenergic receptor (β_1_AR) agonist isoproterenol hydrochloride and inverse agonist ICI118551 hydrochloride were from Sigma-Aldrich. The adenosine A_2A_ receptor (A_2A_R) agonist NECA and antagonist ZM241385 were also from Sigma Aldrich. Serotonin 5HT_1B_ receptor (5HT_1B_R) agonist donitriptan hydrochloride and selective antagonist SB224289 hydrochloride were from Santa Cruz Biotechnology; the agonist sumatriptan succinate was from Cayman chemical. Angiotensin II receptor (AT_1_R) agonist angiotensin II was from Tocris. All radioactive ligands were from PerkinElmer.

### GPCR constructs, expression and purification

#### Human adenosine A_2A_ receptor (A_2a_R)

Two different A_2A_R constructs were used during this work. For SEC experiments using purified receptor, an A_2A_R construct was used that contained an N-terminal thioredoxin fusion protein to increase the molecular weight of the receptor. Without this fusion protein, A_2A_R and the mini-G protein had identical mobility on SDS-PAGE, thus making it difficult to visualise the separate components when analyzing a complex. The thioredoxin-A_2A_R fusion protein consisted of an N-terminal cleavable leader sequence (gp67), His10 tag and TEV protease cleavage site, followed by thioredoxin, which was connected to wild-type human A_2A_R (residues 6–316) through an EAAAKA linker. A_2A_R contained the N154A mutation to remove a potential N-linked glycosylation site. For all other experiments, a C-terminally truncated human A_2A_R construct was used (residues 1–317), which contained a C-terminal His10 tag and TEV protease cleavage site and the N154A mutation to remove the potential N-linked glycosylation site. Both constructs were expressed using the baculovirus expression system as described previously [20, 26]. Cells were harvested by centrifugation 72 hours post infection, resuspended in hypotonic buffer (20 mM HEPES pH7.5, 1 mM EDTA, 1 mM PMSF), flash-frozen in liquid nitrogen and stored at –80°C until use. Purification of the receptor was performed in DDM using Ni^2+^-affinity chromatography followed by SEC essentially as described previously [20, 26].

#### Turkey β_1_-adrenergic receptor (β_1_AR)

A truncated version of wild type turkey β_1_AR (construct βAR6; [27]) contained truncations at the N-terminus and the C-terminus and a C-terminal His6 tag for purification [27], and was expressed using the baculovirus expression system at 27°C as described previously [28]. Cells were harvested by centrifugation 48 hours post infection, resuspended in hypotonic buffer (20 mM Tris-HCl pH8, 1 mM EDTA, 1 mM PMSF), flash-frozen in liquid nitrogen and stored at -80°C until use.

#### Human angiotensin type II receptor 1 (AT_1_R)

Wild type AT_1_R (residues 1–359) had a C-terminal factor X cleavage site followed by GFP and a His10 tag for purification, and was expressed using the tetracycline-inducible mammalian expression system as a stable cell line in HEK293 cells [29]. Cells were grown in DMEM containing 5% tetracycline-free FBS until they were 80% confluent and then tetracycline was added to a final concentration of 1 μg/ml. Cells were grown for 24 hours and then harvested, and resuspended in PBS, flash frozen in liquid nitrogen and stored at -80°C until use.

#### Rat neurotensin receptor (NTSR1)

NTSR1 was expressed as described previously [13]. The baculovirus construct NTSR1 consisted of the hemagglutinin signal peptide and the Flag tag, followed by the wild-type rat NTSR1 (residues 43–396) and a C-terminal His10 tag. Recombinant baculovirus was generated using a modified pFastBac1 transfer plasmid (Invitrogen). *Trichoplusia ni* cells were infected with recombinant virus, and the temperature was lowered from 27°C to 21°C. Cells were harvested by centrifugation 48 hours post infection, resuspended in hypotonic buffer (10 mM HEPES pH 7.5, 10 mM MgCl_2_, 20 mM KCl), flash-frozen in liquid nitrogen and stored at -80°C until use.

#### Human serotonin 5HT_1B_ receptor (5HT_1B_R)

Wild-type 5HT_1B_R (residues 34–390) was modified to contain a C-terminal TEV cleavage site and a His10 tag, cloned into plasmid pBacPAK8 and recombinant baculoviruses were prepared using the FlashBAC ULTRA system (Oxford Expression Technologies). *Trichoplusia ni* cells were grown in ESF921 media (Expression Systems) to a density of 3x10^6^ cells/ml, infected with 5HT_1B_R baculovirus and incubated for 48 h at 27°C for expression. Purification of the receptor was performed in either DDM or LMNG using Ni^2+^-affinity chromatography followed by SEC.

### Expression, purification and stability of G protein subunits

**Fig 2:**
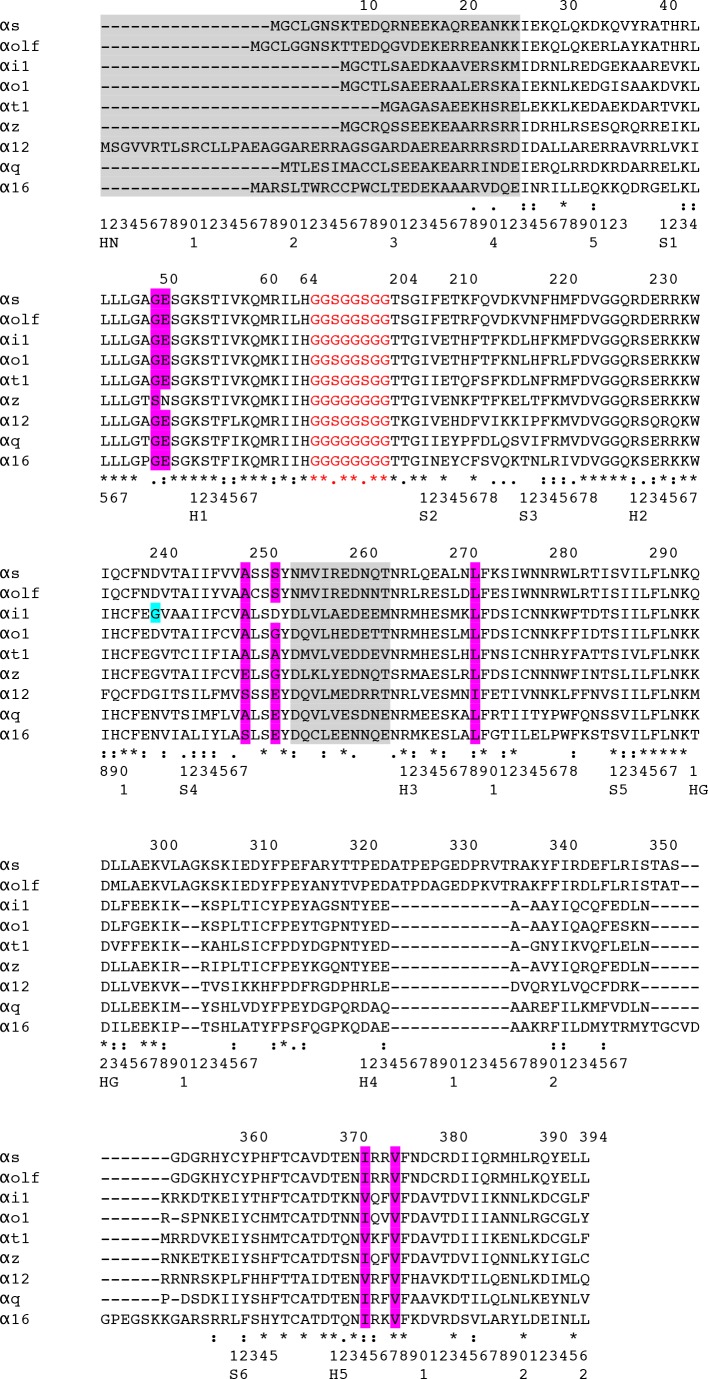
Alignment of Gα GTPase domain protein sequences. The amino acid sequences aligned are of the wild type GTPase domains of the Gα subunits used in this study to create the initial mini-G proteins. The GαAH domain (not shown) was deleted and replaced by a linker (GGGGGGGG or GGSGGSGG in red). To construct mini-G proteins, the residues highlighted in grey were deleted and residues highlighted in magenta were mutated to the following (Gα_s_ residue number and the CGN in superscript): D49^S1H1.3^, N50^S1H1.4^, D249^S4.7^, D252^S4H3.3^, D272^H3.8^, A372^H5.4^, I375^H5.7^. The glycine mutation (G217D, highlighted in cyan) was incorporated into G_i1_ only, to improve expression (see Results & Discussion). Numbering above the sequences is for Gα_s_ and the CGN system below the sequence is used for reference [6].

For constructs see Fig 2 and S1–S3 Figs. Expression, purification and stability measurements by differential scanning fluorimetry (DSF) of the mini-G proteins as well as the non-lipidated Gβ_1_γ_2_ dimer, were performed following the protocols described in [24, 30]. The stability of mini-G proteins was also determined in detergent using native DSF (NanoTemper Prometheus). Mini-G proteins (2 mg/ml) in 50 mM HEPES pH 7.5 (KOH), 20 mM MgCl_2_, 50 mM NaCl, 1 μM GDP were mixed with either no detergent (control), 0.1% LMNG or 0.1% DDM. Samples were incubated on ice (minimum 30 min) prior to heating on the Prometheus (20% excitation, 15°C-85°C, rate of 2.0°C/min) and the onset of scattering determined. The conditions used for the native DSF measurements mimic those used in the formation of a mini-G protein–GPCR complex (contains 1 μM GDP and detergent), which differ from the conditions used for good stability of the GDP-bound state of mini-G proteins (contains 1 mM GDP and no detergent).

### SEC

#### (1) A_2A_R–mini-G_s_ complex

The thioredoxin fusion construct of A_2A_R was purified in DDM essentially as described previously [19]. A_2A_R and mini-G protein were mixed in a 1:1.2 molar ratio, apyrase (0.1 U/ml final concentration) was added and the sample was incubated overnight on ice before loading onto a Superdex S200 10/300 size exclusion column (10 mM HEPES pH 7.5, 100 mM NaCl, 1 mM MgCl_2_, 100 μM NECA, 0.02% DDM; 4°C, 0.5 ml/min). Peak fractions were analysed by SDS-PAGE.

#### (2) 5HT_1B_R–mini-G_o1_ complex

Donitriptan-bound 5HT_1B_R purified in DDM was mixed with mini-G_o1_ in a 1:1.2 molar ratio. Apyrase (0.1 U/ml final concentration) was added and the sample was incubated 4 h on ice before loading onto a Superdex S200 10/300 size exclusion column (20 mM HEPES pH 7.5, 100 mM NaCl, 1 mM MgCl_2_, 1 μM donitriptan, 0.03% DDM; 4°C, 0.5 ml/min). Peak fractions were analysed by SDS-PAGE.

### FSEC assays

#### (1) A_2A_R

Insect cell membranes containing a total of 20 μg (560 pmol) wild-type A_2A_R (20 x 10^6^ cells) were solubilized for 30 min on ice in 40 mM HEPES pH7.5, 500 mM NaCl, 2 mM MgCl_2_, 2 U/mL apyrase (Sigma-Aldrich), and 0.5% (v/v) DDM (final volume of 2 ml, concentration of A_2A_R 280 nM). Insoluble material was removed by ultracentrifugation (30 min, 4°C, 135,000 x*g*). The supernatant was divided into aliquots for the subsequent assay. To 900 μl of the supernatant was added either the agonist NECA or the inverse agonist ZM241385 (negative control), both at a final concentration of 60 μM. GFP-mini-G_s_ (6 μg; 110 pmol, final concentration 122 nM) was then added and allowed to bind for 90 min on ice before loading 200 μl onto a Superdex S200 10/300 size exclusion column (buffer 20 mM HEPES pH 7.5, 100 mM NaCl, 10 mM MgCl_2_, 1 μM NECA or ZM241385, 0.03% DDM, 4°C, flow rate 0.45 ml/min). The control sample contained 6 μg GFP-mini-G_s_ only in 500 μl assay buffer. GFP fluorescence was detected by a Hitachi fluorometer (mV) set to an excitation of 488 nm and an emission of 525 nm.

#### (2) β_1_AR

Insect cell membranes containing a total of 8 μg (178 pmol) wild-type β_1_AR (30 x 10^6^ cells) were solubilized for 30 min on ice in 20 mM Tris-HCl pH8, 500 mM NaCl, 5 mM MgCl_2_, 2 U/mL apyrase and 0.5% (v/v) DDM (final volume 2 ml, β_1_AR concentration 90 nM). Insoluble material was removed by ultracentrifugation (30 min, 4°C, 135,000 x*g*). The supernatant was divided into aliquots for the subsequent assay. Isoprenaline (100 μM final concentration) or ICI118551 (10 μM final concentration) were added to 500 μl of the supernatant. GFP-mini-G_s_ (6 μg, 110 pmol) was then added to give a final concentration of 122 nM and allowed to bind for 90 min on ice before loading 200 μl onto a Superdex S200 10/300 size exclusion column (buffer 20 mM HEPES pH 7.5, 100 mM NaCl, 10 mM MgCl_2_, 1 μM isoprenaline or ICI118551, 0.03% DDM, 4°C, flow rate 0.45 ml/min). The control sample contained 6 μg GFP-mini-G_s_ only in 500 μl assay buffer.

#### (3) 5HT_1B_R

When detergent-solubilized unpurified receptor was used, insect cells expressing 610 pmol 5HT_1B_R (40 x 10^6^ cells) were resuspended in 20 mM HEPES pH 7.5, 100 mM NaCl, 10 mM MgCl_2_, 2 U/ml apyrase to a final cell density of 20 x 10^6^ cells/ml and solubilized with 0.5% DDM (45 min, 4°C, final volume 2 ml, 5HT_1B_R concentration 305 nM). Insoluble material was removed by ultracentrifugation (30 min, 4°C, 135,000 x*g*). The supernatant was divided into 900 μl aliquots for the subsequent assay. GFP-mini-G_o1_ (5 μg, 100 pmol) was added (final concentration 111 nM) with either donitriptan or SB224289, each to a final concentration of 100 μM, and allowed to bind for 90 min on ice before loading 500 μl onto a Superdex S200 10/300 size exclusion column. The control sample contained 5 μg GFP-mini-G_o1_ in 500 μl assay buffer.

In some FSEC experiments, purified 5HT_1B_R was used. Donitriptan-bound, purified receptor (120 μg; 3 nmol) in either LMNG or DDM was incubated for 90 min on ice with 4 μg (60–80 pmol) either of GFP-mini-G_i1,_ GFP-mini-G_o1_ or GFP-mini-G_s_ (negative control) in a final volume of 450 μl. Samples (200 μl) were then loaded onto Superdex S200 10/300 size exclusion column (buffer 20 mM HEPES pH 7.5, 100 mM NaCl, 10 mM MgCl_2_, 1 μM donitriptan, 0.03% DDM or 0.001% LMNG buffer, 4°C, flow rate 0.45 ml/min). The final concentration of purified 5HT_1B_R was 6.7 μM and the final concentrations of the mini-G proteins were as follows: GFP-mini-G_s/i1_, 133 nM; GFP-mini-G_i1_, 167 nM; GFP-mini-G_o1_, 170 nM; GFP-mini-G_s_, 167 nM; GFP-mini-G_i1_β_1_γ_2_, 90 nM.

### Fluorescent saturation binding assay (FSBA)

#### (1) β_1_AR

Membranes prepared from insect cells expressing β_1_AR (50 x 10^6^ cells) were solubilized in 20 mM Tris-HCl pH8, 500 mM NaCl, 3 mM imidazole, 2 U/ml apyrase 0.5% DDM (1 hour, 4°C, final volume 8 ml, β_1_AR concentration 37.5 nM). Insoluble material was removed by ultracentrifugation (30 min, 4°C, 135,000 x*g*) and the supernatant was divided into two aliquots. The agonist isoprenaline was added to one sample (final concentration 10 μM) and the inverse agonist ICI118551 was added to the other (final concentration 1 μM). Samples were then aliquoted 200 μl per well into a black Ni^2+^-coated 96-well plate (Pierce; Thermo Fisher). The receptor was allowed to bind *via* its His tag for 1 h on ice. The supernatant was then aspirated and 200 μl GFP-mini-G_s_ at varying concentrations (0 to 2.8 μM) were added and incubated for a further 90 min on ice. The supernatant was then removed by aspiration and each well washed 4 times with buffer A (10 μM isoprenaline (agonist), 20 mM Tris-HCl pH8, 100 mM NaCl, 1 mM MgCl_2_, 1 mg/mL BSA, 30 mM imidazole, 0.03% DDM,) or buffer B (1 μM ICI118551 (inverse agonist), 20 mM Tris-HCl pH8, 100 mM NaCl, 1 mM MgCl_2_, 1 mg/mL BSA, 30 mM imidazole, 0.03% DDM). Elution of the receptor–GFP-mini-G_s_ complex from the sides of the well to make a homogeneous solution was performed with 200 μl of the respective wash buffers that contained 300 mM imidazole. Fluorescence was then measured using a Pherastar plate reader (BMG Labtech, Inc.) with excitation at 485 nm and emission at 520 nm. ΔF data (fluorescence agonist condition minus fluorescence antagonist condition) corresponding to specific binding were analysed by non-linear regression using GraphPad Prism version 5.0 (GraphPad Software, San Diego, CA) and apparent *K*_*D*_ values derived from one site-specific binding analysis.

#### (2) A_2A_R

The assay was performed essentially as described above for β_1_AR, but the buffer conditions were different. Solubilisation of insect cell membranes (40 x 10^6^ cells) was performed in 10 ml of 20 mM Tris-HCl pH8, 500 mM NaCl, 10 mM imidazole, 2 U/ml apyrase and 0.5% DDM (1 h, 4°C, final volume 10 ml, A_2A_R concentration 112 nM). After ultracentrifugation, the agonist NECA (10 μM final concentration) was added to one supernatant sample and the inverse agonist ZM241385 (10 μM final concentration) to the other. Washing buffers for A_2A_R were buffer C (10 μM NECA, 20 mM Tris-HCl pH8, 100 mM NaCl, 1 mM MgCl_2_, 1 mg/mL BSA, 50 mM imidazole, 0.03% DDM) or buffer D (10 μM ZM241385, 20 mM Tris-HCl pH8, 100 mM NaCl, 1 mM MgCl_2_, 1 mg/mL BSA, 50 mM imidazole, 0.03% DDM).

#### (3) 5HT_1B_R

Insect cells expressing 5HT_1B_R (50 x 10^6^ cells) were solubilized with buffer containing 10 μM Donitriptan, 20 mM Tris-HCl pH8; 500 mM NaCl; 10 mM imidazole, 2 U/ml apyrase and 0.5% DDM (1 h, 4°C, final volume 6 ml, 5HT_1B_R concentration 127 nM). Insoluble material was removed by ultracentrifugation (30 min, 4°C, 135,000 x*g*) and 200 μl of supernatant was then aliquoted per well into a black Ni^2+^-coated 96-well plate. The receptor was allowed to bind *via* its His tag for 1 h on ice. The supernatant was then aspirated and 200 μl either of GFP-mini-G_o1,_ GFP-mini-G_s/i1_ or GFP-mini-G_s_ (negative control) at varying concentrations (from 0 to 5 μM) were added and incubated for a further 90 min on ice. The supernatant was then removed by aspiration and each well washed 4 times with buffer E (1 μM Donitriptan, 20 mM Tris-HCl pH8, 100 mM NaCl, 1 mM MgCl_2_, 1 mg/mL BSA, 50 mM imidazole, 0.03% DDM). Elution was carried out with 200 μL of buffer E containing 300 mM imidazole. ΔF data (fluorescence G_o1_ condition minus fluorescence G_s_ condition) corresponding to specific binding were analysed by non-linear regression using GraphPad Prism version 5.0 (GraphPad Software, San Diego, CA) and apparent *K*_*D*_ values derived from one site-specific binding analysis.

### Competition binding assay

Insect cells expressing 5HT_1B_R were resuspended in 1 ml of assay buffer (20 mM HEPES pH7.5, 100 mM NaCl, 1 mM MgCl_2_, 1 mM ascorbate, 20 μM pargyline) at a final concentration of 2 x 10^6^ cells/ml. Cells were sheared by 10 passages through a bent 26G needle. The supernatant was diluted 10-fold in assay buffer and aliquots (900 μl) taken for each sample. Mini-G protein (100 μl, 25 μM final concentration) or buffer (negative control) was added. The mixture was aliquoted into a 0.2 ml PCR plate, 96 μl per well. Sumatriptan (12 μl), prepared in assay buffer also containing 2 U/ml apyrase, was added to each well (final concentrations in the range of 100 pM to 1 mM). Non-specific binding was determined in the presence of 100 μM donitriptan. Samples were mixed and incubated at 4°C for 2 h. [^3^H]-GR125743 (12 μl) was added at its apparent *K*_*D*_ (10 nM) concentration. Samples were mixed and incubated at 4°C for 2 h before filtering through 96-well glass fibre GF/B filter plate (Merck Millipore) and washing with ice-cold assay buffer. Filters were dried, punched into scintillation vials and 4 ml Ultima Gold scintillant (Perkin Elmer) were added. Radioactivity was quantified by scintillation counting (1 min per sample) using a Tri-Carb counter (Perkin Elmer), and *K*_*i*_ values were determined using GraphPad Prism version 5.0 (GraphPad Software, San Diego, CA)

### Thermostability assay

#### (1) A_2A_R

Membranes from *Trichoplusia ni* cells expressing wild-type human A_2A_R were resuspended in *T*_*m*_ buffer (25 mM HEPES pH 7.5, 100 mM NaCl, 1 mM MgCl_2_) and homogenized by ten passages through a 26G needle. Mini-G protein was added at a final concentration of 25 μM. ^3^H-NECA and unlabeled NECA, prepared in assay buffer containing 2 U/ml apyrase, were mixed in a molar ratio of 1:5 and added to the membranes to give a final concentration of 1 μM (approximately ten-fold above the apparent *K*_*D*_). The samples were incubated at room temperature for 1 h, then chilled on ice for 30 min. Decylmaltoside (DM) was added to a final concentration of 0.13%, and samples were incubated on ice for 1 h. Cell debris and insoluble material were removed by centrifugation (5 min, 20,000 x*g*, 4°C) and the supernatant was aliquoted (120 μl) into PCR strips. Samples were heated to the desired temperature for exactly 30 min, then quenched on ice for 30 min. Samples (50 μl) were loaded onto gel-filtration resin (Toyopearl HW-40F) packed into a 96-well filter plate (Millipore), which was centrifuged to separate receptor-bound from free radioligand [31]. Nonspecific binding was determined in the presence of 200 μM unlabelled NECA. Radioactivity was quantified by liquid scintillation counting using a MicroBeta TriLux scintillation counter (PerkinElmer). Data were analysed by nonlinear regression using GraphPad Prism software. Apparent *T*_*m*_ values were derived from sigmoidal dose-response analysis performed by non-liner regression. Results represent the mean ± SEM of two independent experiments, performed in duplicate.

#### (2) NTSR1

Cell pellets from 10 ml of insect cell cultures were resuspended in 1.8 ml buffer containing DDM to give a final buffer composition of 50 mM Tris-HCl pH 7.4, 100 mM NaCl, 1 mM MgCl2, 1% (w/v) DDM. The samples were placed on a rotating mixer at 4°C for 1 hour. Cell debris and non-solubilized material were removed by ultracentrifugation (152,800 x*g*, 4°C, 30 min), and the supernatant containing detergent-solubilized NTSR1 was used to test for thermal stability in the presence of NTS and mini-G proteins. For thermal denaturation curves, the supernatants were diluted 6.67-fold into assay buffer (50 mM Tris-HCl pH 7.4, 100 mM NaCl, 1 mM MgCl_2_) containing 22.5 μM mini-G protein and 10 nM ^3^H-NTS and incubated for 1 hour on ice. After addition of apyrase (0.25 units/ml, NEB), the sample was placed on ice for an additional 30 min. Samples (120 μl aliquots) were exposed to different temperatures between 0°C and 60°C for 30 min and placed on ice. Separation of receptor–ligand–mini-G protein complex from free ^3^H-NTS (100 μl) was achieved by centrifugation-assisted gel filtration (spin assay) using Bio-Spin 30 Tris columns (BioRad), equilibrated with RDB buffer [50 mM Tris-HCl pH7.4, 1 mM EDTA, 0.1% (w/v) DDM, 0.2% (w/v) CHAPS, 0.04% (w/v) CHS], essentially as described previously [32]. Control reactions on ice were recorded at the start and at the end of each denaturation experiment. The percentage of activity remaining after heat exposure was determined with respect to the unheated control. Data were analyzed by nonlinear regression using a Boltzmann sigmoidal equation in the Prism software (GraphPad).

#### (3) AT_1_R

HEK 293 cells expressing wild type AT_1_R were resuspended in a radioligand binding assay buffer (50 mM HEPES pH 7.4, 150 mM NaCl, 1 mM EDTA, 0.1% BSA, 40 μg/ml bacitracin) and homogenized by sonication (4 sec pulse). Mini-G protein and apyrase were added at a final concentration of 25 μM and 0.1 U/ml, respectively. ^125^I-Ang II and unlabeled Ang II were added at a concentration of 0.5 nM and 25 nM respectively (approximately 50 times the apparent *K*_D_ value). The sample was incubated at room temperature (20°C) for an hour, chilled on ice for 10 minutes and then digitonin was added to a final concentration of 1% and incubated on ice for an hour. Insoluble material was removed by centrifugation (2 min, 20,000 x*g*, 4°C). The reaction mix was split into a number of 115 μl aliquots and each was incubated at various temperatures for exactly 30 minutes. The reactions were then quenched on ice for 5 minutes. ^125^I-Ang II bound to AT_1_R was separated from unbound ^125^I-Ang II using centrifugation-assisted gel filtration column, essentially as described previously [29]. Non-specific binding was determined using a 500-fold excess of cold ligand. Radioactivity was measured using liquid scintillation counting. Data was analysed by non-linear regression using GraphPad prism software and apparent *T*_m_ values were derived by non-linear regression of the sigmoidal dose-response curve.

## Results and discussion

### Initial development of new mini-G proteins

The recently designed minimal G protein, mini-G_s_ [24], comprises only the GαGTPase domain from G_s_ and 3 deletions and 7 mutations to thermostabilise it (Fig 2). Mini-G_s_ coupled to both the β_1_-adrenergic receptor (β_1_AR) and the adenosine A_2A_ receptor (A_2A_R), and resulted in the same increase in agonist affinity as observed for heterotrimeric G_s_ coupling [20, 24]. However, there are 4 families of Gα subunits (Fig 1) and GPCRs couple to distinct G proteins depending upon their physiological function [25]. Therefore, to provide tools for the structure determination of GPCRs in their fully active state, it was necessary to develop versions of mini-G proteins for at least one member from each of the other families. All of the mutations and deletions used to create mini-G_s_ are located within conserved regions of the Gα subunit (Fig 2). Therefore, in theory, these mutations were potentially transferable to the other Gα families, allowing the production of a panel of mini-G proteins capable of coupling to any GPCR.

Archetypical members from each Gα family were selected and include the following: G_olf_ from the G_s_ family, G_i1_, G_o1_, G_z_ and G_t_ from the G_i_ family, G_q_ and G_16_ from the G_q/11_ family, and G_12_ from the G_12/13_ family. The mutations required to convert Gα_s_ into mini-G_s_ were transferred *en bloc* to the selected Gα proteins to produce a mini-G protein version of each (Fig 2). These mutations were the following: (i) deletion of all amino acid residues N-terminal of Ile/Leu^HN43^; (ii) deletion of the α-helical domain between residues H^H1S2.12^ and the Thr, three residues N-terminal to Ile^S2.1^, and replacement with an 8 amino acid residue linker; (iii) deletion of 10 amino acid residues of switch III between Tyr^S4H3.4^ and Asn/Ser^S4H3.15^; (iv) mutating 7 residues to D49^S1H1.3^, N50^S1H1.4^, D249^S4.7^, D252^S4H3.3^, D272^H3.8^, A372^H5.4^, I375^H5.7^. Residue numbers are for Gα_s_ and superscripts refer to the CGN system for comparing residues in G proteins [6]. Initial characterization of each mini-G protein was performed by assessing expression in *Escherichia coli* and purification by Ni^2+^-affinity chromatography and size exclusion chromatography (SEC). Four out of the eight engineered mini-G proteins (mini-G_olf_, mini-G_i1_, mini-G_o1_ and mini-G_12_) fulfilled these initial criteria *i*.*e*. they were all stable enough in their basal conformation to allow high-yield expression and purification. The yield of purified mini-G protein per litre of culture and their stability as measured by differential scanning fluorimetry (in parentheses) are as follows: mini-G_s_, 100 mg/L (65°C); mini-G_olf_, 80 mg/L (65°C); mini-G_o1_ 100 mg/L (64°C); mini-G_12_ 25 mg/L (73°C). The worst expressed of the four new mini-G proteins was mini-G_i1_, so an additional mutation G217D was incorporated and the truncation at the N-terminus shortened, which increased the yield of pure protein to 12 mg/L, although the stability was only 48°C. Thus, mini-G_olf_, mini G_i1_, mini-G_o1_ and mini-G_12_ were all of sufficient stability to be used to test their ability to couple to relevant GPCRs. The amino acid sequences of the mini-G proteins are given in S1 Fig.

Four mini-G proteins were not expressed in *E*. *coli*, namely mini-G_z_, mini-G_t_, mini-G_16_ and mini-G_q_ (amino acid sequences are given in S2 Fig). The failure of the *en bloc* transfer of the deletions and mutations from mini-G_s_, despite the high conservation of G protein structures, highlights our lack of understanding of the folding of these proteins. Indeed, it is well known that an accessory factor, Ric8, is required for the efficient folding of G_q_ in mammalian cells [33], and other unknown factors may also be required. For the purpose of this study, we therefore did not perform any further development of mini-G_t1_ and mini-G_z_, given that two other members of the G_i_ family, mini-G_i1_ and mini-G_o1_, already gave stable mini-G proteins. In contrast, as neither member of the G_q_ family tested produced a stable mini-G protein, we decided to develop alternative strategies to make a usable version of mini-G_q_, whilst further work on mini-G_16_ was terminated. The successful engineering of a version of mini-G_q_ chimera will be discussed later.

### Assay development and validation using the mini-G_s_ system

The ultimate goal of developing mini-G proteins is the structure determination of GPCRs in the fully active state bound to an agonist and a mini-G protein. In the simplest format, this necessitates the purification of the GPCR in detergents and forming the G protein–GPCR complex from the purified components *in vitro*. It was therefore essential to devise some simple assays that could assess whether a mini-G protein had coupled to a GPCR in detergent solution. This turned out to be not as straightforward as originally anticipated due to the potential instability of either the GPCR and/or mini-G protein in either their inactive and/or active conformations. These issues were not obvious when the original work on the development of mini-G_s_ was performed, because mini-G_s_ is one of the most stable mini-G proteins developed and also the thermostabilised β_1_-adrenergic receptor (β_1_AR) and the wild type adenosine A_2A_ receptor (A_2A_R) were both much more stable than other GPCRs. We therefore developed five separate assays for assessing whether a mini-G protein coupled to a GPCR and/or formed a stable complex in detergent. These were all first tested using mini-G_s_ coupling to β_1_AR and A_2A_R. Each assay has its own limitations, which are often apparent in the subsequent sections where they were used on less stable receptors and the newly developed mini-G proteins, and these are discussed below. The five different assays that were used are the following: (i) agonist affinity shift assay; (ii) thermostability assay (TSA); (iii) fluorescence-based saturation binding analysis (FSBA) of GFP-mini-G protein binding; (iv) fluorescence-detection size exclusion chromatography (FSEC); (v) size exclusion chromatography (SEC) of purified complex. A brief rationale for the use of each assay with their advantages and disadvantages are given below.

#### (i) Agonist affinity shift assay

The development of mini-G_s_ relied on the agonist affinity shift assay to identify those mutants that coupled to β_1_AR [24]. It is generally considered that the defining feature of G protein coupling is an increase in the affinity of an agonist for the G protein–GPCR complex compared to the GPCR alone. For example, wild type β_2_AR binds an agonist 100-fold more tightly when coupled to a G protein than the receptor alone [34]. However, the shift in agonist affinity in other receptors is often considerably smaller than that observed for β_2_AR, such as the 10-fold shift in agonist affinity observed in β_1_AR [34]. However, the advantage of this assay is that it can be performed using standard pharmacological procedures in high-throughput, using receptors in either membrane preparations or solubilized in detergent. Assays may use either a radiolabelled agonist in saturation binding experiments or, more usually, a radiolabelled antagonist in competition binding experiments [20, 24] (and see experiments below on the serotonin 5HT_1B_ receptor). The advantage of this assay is that it is very sensitive and can be performed on membrane-bound receptors *i*.*e*. in a format where the receptor is most stable in all conformations. The disadvantage of this assay is that it requires suitable radioligands for the receptor under study and these are not readily available for all GPCRs.

#### (ii) Thermostability assay

The thermostability of a detergent-solubilised GPCR depends upon the type of detergent used and whether the receptor is either ligand-free, agonist-bound or antagonist-bound [35, 36]. In addition, the receptor stability tends to be increased by an increase in affinity and/or decrease in the off-rate of the ligand [37]. Often, the agonist bound state is one of the least stable conformations of a receptor, presumably because agonists increase the probability of transitions to a fully active state. In the inactive state there is close packing of the intracellular surface of the transmembrane α-helices. Upon activation, the outward movement of helices 5 and 6 disrupts this close packed structure and creates a crevice where the C-terminus of the G protein binds, thus allowing G protein coupling [38]. The structures of non-rhodopsin GPCRs in the fully active state have been determined only when they have been stabilized through binding of a heterotrimeric G protein [2], a conformation-specific nanobody [15, 18, 19] or a mini-G protein [20]. The interface between a GPCR and a G protein is over 1000 Å^2^ [2, 20], and is therefore predicted to increase the thermostability of the agonist-bound GPCR–G protein complex compared to the agonist-bound GPCR. This was observed for both β_1_AR and A_2A_R, which were consistently more stable in the agonist-bound state when coupled to mini-G_s_ in a variety of different detergents compared to when mini-G_s_ was absent [20, 24].

A typical thermostability assay measures how much of a radiolabelled agonist remains bound to a detergent-solubilised receptor after heating at different temperatures for 30 minutes [36]. The advantage of this assay is that it is fast and high-throughput and can be performed in any detergent of choice. Another advantage is that the agonist–GPCR–mini-G protein complex can be pre-formed in membranes, which may stabilise the receptor upon detergent solubilisation. If there is a shift in thermostability in the presence of a mini-G protein, then this is strongly suggestive of binding or coupling.

#### (iii) Fluorescence-detection size exclusion chromatography (FSEC)

**Fig 3:**
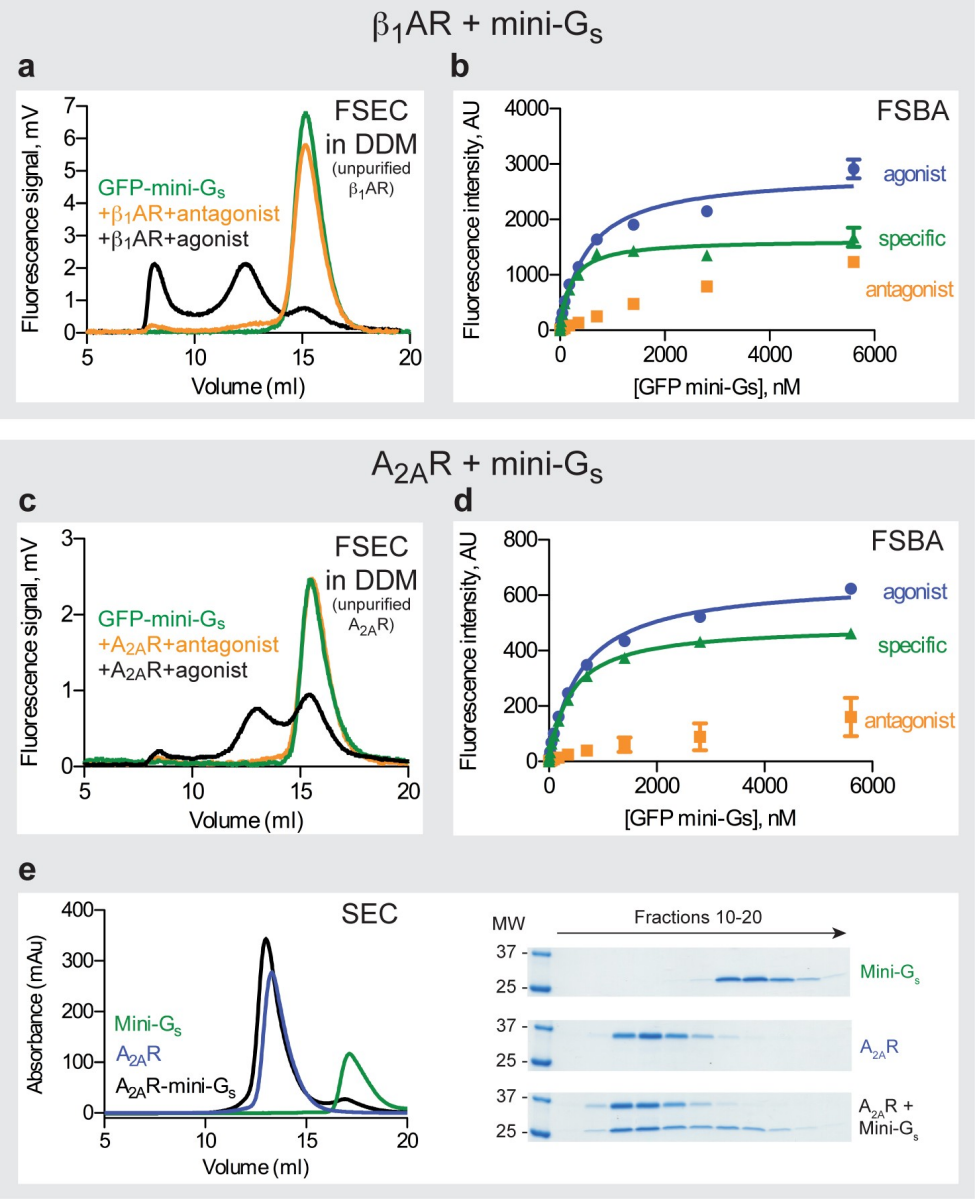
The β_1_AR−mini-G_s_ and A_2A_R−mini-G_s_ complexes. (**a**) FSEC traces of GFP-mini-G_s_ with β_1_AR (retention volumes are given in parentheses): green, GFP-mini-G_s_ (15.1 ml); orange, GFP-mini-G_s_ with β_1_AR bound to the inverse agonist ICI118551 (15.1 ml); black, GFP-mini-G_s_ with β_1_AR bound to the agonist isoprenaline (8 ml, 12.1 ml and 15.1 ml). Representative chromatograms from at least two independent experiments are shown. (**b**) Measurement of GFP-mini-G_s_ affinity to DDM-solubilized β_1_AR using a fluorescent saturation binding assay (FSBA); blue circles, β_1_AR bound to the agonist isoprenaline (total binding); orange squares, β_1_AR bound to the inverse agonist ICI118551 (non-specific binding); green triangles, specific binding, with an apparent *K*_*D*_ of 200 ± 1 nM (mean ± SEM, n = 2). Curves shown are from a representative experiment. (**c**) FSEC traces of GFP-mini-G_s_ with DDM-solubilised A_2A_R (retention volumes are given in parentheses): green, GFP-mini-G_s_ (15.1 ml); orange, GFP-mini-G_s_ with A_2A_R bound to the inverse agonist ZM241385 (15.1 ml); black, GFP-mini-G_s_ with A_2A_R bound to the agonist NECA (12.5 ml and 15.1 ml). Representative chromatograms from at least two independent experiments are shown. (**d**) Measurement of mini-G_s_ affinity to DDM-solubilized A_2A_R using FSBA: blue circles, A_2A_R bound to the agonist NECA (total binding); orange squares, A_2A_R bound to the inverse agonist ZM241385 (non-specific binding); green triangles, specific binding, with an apparent *K*_*D*_ of 430 ± 24 nM (mean ± SEM, n = 2). (**e**) Analytical size exclusion chromatography (SEC) of mini-G_s_ bound to purified A_2A_R (retention volumes are given in parentheses): black, A_2A_R–mini-G_s_ complex, 153 kDa (13 ml); blue, A_2A_R, 133 kDa (13.3 ml); green, mini-G_s_, 22 kDa (17.2 ml). Three panels to the right of the SEC traces are coomassie blue-stained SDS-PAGE gels of fractions from 3 separate SEC experiments: top panel, mini-G_s_; middle panel, A_2A_R; bottom panel, mini-G_s_ mixed with NECA-bound A_2A_R (1.2:1 molar ratio).

FSEC is a rapid methodology for assessing whether a membrane protein fused to GFP is stable in detergent by performing SEC on an unpurified detergent solubilisate and monitoring GFP fluorescence in the eluate [39]. A membrane protein stable in detergent gives a symmetrical peak at a size consistent with the molecular weight of the membrane protein plus the mass of specifically bound detergent and lipid. By fusing GFP to the N-terminus of mini-G proteins (S3 Fig), it was possible to use FSEC to monitor whether a stable complex was formed between the mini-G protein and a GPCR. The GFP-mini-G_s_ fusion protein has a molecular weight of 54 kDa and migrated with a retention volume of 15.1 ml on FSEC. When this was mixed with either unpurified DDM-solubilised β_1_AR or A_2A_R in the presence of an agonist, then an additional peak was observed at 12.1–12.5 ml (Fig 3A and 3C), which was consistent with the molecular weight of the detergent-solubilised receptor bound to GFP-mini-G_s_ (~180 kDa). This additional peak was not observed if the receptors were bound to an inverse agonist. An additional peak was sometimes observed at a retention volume of 8 ml, which corresponds to the void volume of the SEC column and was due presumably to aggregates of GFP-mini-G_s_.

The advantage of this assay is that it is a quick assessment of whether a GPCR forms a complex with a mini-G protein, because the receptor does not need to be purified and the SEC experiment takes under an hour. However, a major limitation is that only small amounts of GFP-mini-G_s_ can be used per experiment to avoid saturation of the detector and producing a very broad peak that would obscure the presence of the complex between the GPCR and GFP-mini-G protein. An additional limitation is the amount of membranes that can be solubilized efficiently using 0.5% DDM (to ensure maximal stability of mini-G_s_; see below) and maintaining the small volume (500 μl) required for loading on the FSEC column (24 ml). Thus in the data shown (Fig 3A and Fig 3C) the amount of receptor is limiting and only a proportion of the GFP-mini-G_s_ is bound to receptor and is shifted in apparent molecular weight. In addition, the receptor–mini-G protein complex must be detergent-stable for a peak to be observed. Many GPCR–G protein complexes are too unstable to be observed in DDM and therefore it is essential to assess milder detergents such as LMNG (see section on the serotonin 5HT_1B_ receptor).

#### (iv) Fluorescence-based saturation binding analysis of mini-G protein binding

To determine the affinity of mini-G protein binding to a receptor, the fluorescence-based saturation binding assay (FSBA) was developed. In this assay, the amount of the GFP-mini-G protein specifically bound to an immobilized receptor was determined using a fluorescent plate reader. As proof of principle, DDM-solubilized β_1_AR or A_2A_R were immobilized onto Ni^2+^-coated wells of a 96-well plate *via* their C-terminal poly-histidine tag, in the presence of either an agonist or inverse agonist. GFP-mini-G_s_ was then added at increasing concentrations. After washing to remove any non-specifically bound GFP-mini-G_s_, the amount of GFP-mini-G_s_ fluorescence was measured (Fig 3B and 3D). GFP-mini-G_s_ showed a specific saturated binding to the receptor with apparent *K*_*D*_ values of 200 ± 1 nM (n = 2) and 430 ± 24 nM (n = 2) for GFP-mini-G_s_ binding to β_1_AR and A_2A_R, respectively.

The FSBA is a simple assay for determining the affinity of mini-G protein binding to a receptor *in vitro*. However, it must be appreciated that the apparent affinity determined may be specific only for the conditions in the assay. In particular, the type of detergent used may have a profound effect on the affinity, especially if it slightly destabilizes the active state of the receptor. The agonist may also affect the apparent affinity of the mini-G protein, depending on how effective the agonist is in stabilizing the active state of the receptor. However, the FSBA remains a useful tool for biophysical analyses of mini-G protein binding to a receptor.

#### (v) Size exclusion chromatography (SEC)

The ultimate biochemical assay for observing coupling of mini-G proteins to a receptor is combining the purified components *in vitro* and then observing the co-elution of the relevant proteins on SEC [24]. Purified A_2A_R and purified mini-G_s_ were mixed at a molar ratio of 1:1.2 in the presence of the agonist NECA, the complex allowed to form and then separation was performed by SEC. The A_2A_R–mini-G_s_ complex resolved as a predominant peak with an apparent molecular weight of 153 kDa compared with 133 kDa for the receptor alone and 22 kDa for mini-G_s_ alone. SDS-PAGE analysis confirmed the presence of both A_2A_R and mini-G_s_ in fractions from the 153 kDa complex (Fig 3E).

The advantage of using purified components and SEC for analyzing complex formation is that complex formation is observed unambiguously. The conditions for complex formation can be refined and the stability of the complex can be assessed readily after a period of days by repeating the SEC. These data are essential for successful determination of the structure of a GPCR–mini-G protein complex. The disadvantage of this assay is that sufficient quantities of purified receptor are required and this may be limiting in the initial stages of a project. The limitation of the amount of purified receptor required for SEC may be partially overcome by analyzing complex formation using FSEC (see section on 5HT_1B_R).

### Characterisation of mini-G proteins

#### Mini-G_olf_ couples and stabilizes A_2A_R

**Fig 4:**
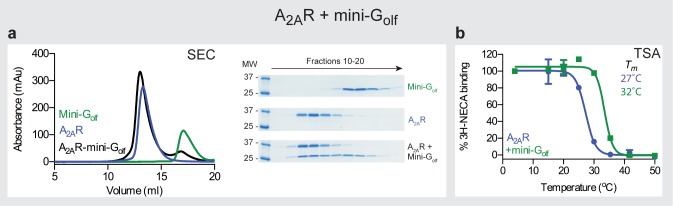
The A_2A_R−mini-G_olf_ complex. (**a**) Analytical SEC of mini-G_olf_ bound to purified A_2A_R (retention volumes are given in parentheses): black, A_2A_R–mini-G_olf_ complex, 153 kDa (13 ml); blue, A_2A_R, 133 kDa (13.3 ml); green, mini-G_olf_, 23 kDa (17.1 ml). Three panels to the right of the SEC traces are coomassie blue-stained SDS-PAGE gels of fractions from 3 separate SEC experiments: top panel, mini-G_olf_; middle panel, A_2A_R; bottom panel, mini-G_olf_ mixed with NECA-bound A_2A_R (1.2:1 molar ratio). (**b**) Thermostability assay (TSA) of unpurified DM-solubilized, ^3^H-NECA-bound A_2A_R. Data were analysed by nonlinear regression and apparent *T*_*m*_ values were determined from analysis of the sigmoidal dose-response curves fitted. *T*_*m*_ values represent mean±SEM of two independent experiments, each performed in duplicate: blue circles, no mini-G_olf_ (27 ± 0.3°C); green squares, mini-G_olf_ (33 ± 1°C). Curves shown are from a representative experiment.

The GTPase domains of G_olf_ and G_s_ share 87% sequence identity (80% for the full length α subunits) and both G proteins couple to A_2A_R [40]. Of the 17 amino acid residues in mini-G_s_ that make direct contact to residues in A_2A_R in the crystal structure of the A_2A_R–mini-G_s_ complex [20], all of these residues are identical except that two Arg residues in G_s_ are replaced with two Lys residues in G_olf_. Despite the high degree of sequence homology between these two isoforms, Gα_olf_ is far more difficult to overexpress than Gα_s_, in fact, the only method reported to produce functional Gα_olf_ is co-expression with the molecular chaperone RIC8B in insect cells [41]. Therefore, we constructed mini-G_olf_ to investigate whether the mini-G protein version would be better expressed that native α subunit. Mini-G_olf_ was constructed by transferring the 7 point mutations and 3 deletions from mini-G_s_ (Fig 2) and mini-G_olf_ was highly expressed in *E*. *coli* and as stable as mini-G_s_. The coupling of mini-G_olf_ to A_2A_R was assessed by SEC of the complex assembled *in vitro* from purified proteins and a thermostability assay [20, 24]. Purified NECA-bound A_2A_R was mixed with mini-G_olf_ and analysed by SEC and SDS-PAGE (Fig 4A). The apparent molecular weight of mini-G_olf_ was 23 kDa (17.1 ml; theoretical molecular weight 26 kDa) and the apparent molecular weight of purified A_2A_R in DM was 133 kDa (13.3 ml). The complex A_2A_R–mini-G_olf_ resolved as a predominant peak with an apparent molecular weight of 153 kDa (13 ml) and contained both A_2A_R and mini-G_olf_. Mini-G_olf_ also stabilized agonist-bound DM-solubilised A_2A_R, with mini-G_olf_-coupled A_2A_R showing an apparent *T*_*m*_ of 32 ± 1°C in comparison with 27 ± 0.3°C for the receptor alone (Fig B). This stability was similar to that obtained with mini-G_s_ (33°C) under the same conditions [20].

The results with mini-G_olf_ were very encouraging in terms of both the transferability of the mutations, the expression and stability of the mini-G_olf_ and the stability of the A_2A_R–mini-G_olf_ complex. Thus where there is a high degree of homology between G proteins, then there is good transferability of the mutations, as was previously observed for the transfer of thermostabilising mutations between GPCRs [42]. These data also suggested that even if the native α subunit is poorly expressed the mini-G protein version may be highly expressed and very stable.

#### Development of chimeric mini-G_s/q_ to study G_q_-coupled receptors

The expression of mini-G_q_ in *E*. *coli* was unsuccessful. One possibility to explain this is that efficient folding of G_q_ *in vivo* is dependent on the molecular chaperone Ric8 [33] and that mini-G_q_ had a similar requirement. Indeed, co-expression of Ric8 with mini-G_q_ in the baculovirus expression system led to the overproduction of mini-G_q_. However, upon purification of mini-G_q_ it was not possible to dissociate Ric8 (results not shown), suggesting that the mini-G_q_ was perhaps not correctly folded and/or was very unstable. Given the lack of success in transferring the mini-G protein mutations from G_s_ to G_q_, another strategy was developed.

The second strategy used to try and develop mini-G_q_ was to transfer the specificity determinants of G_q_ onto mini-G_s_. It is well established that the C-terminal region of a Gα subunit forms the main receptor binding site [43] and is one of the main determinants of coupling specificity [44, 45]. Mutating as few as 3–5 amino acids at the C-terminus of the G alpha subunit has been shown to switch the specificity of coupling to some GPCRs [44, 45]. However, the two GPCR–G protein structures published to date [2, 20] revealed an extensive interface between the receptors and Gα, suggesting that other regions of the G protein may also play a role in specificity. Recent *in vivo* FRET studies suggest that residues within the α5 helix, but distal to the five C-terminal residues, strongly influence specificity [46].

**Fig 5:**
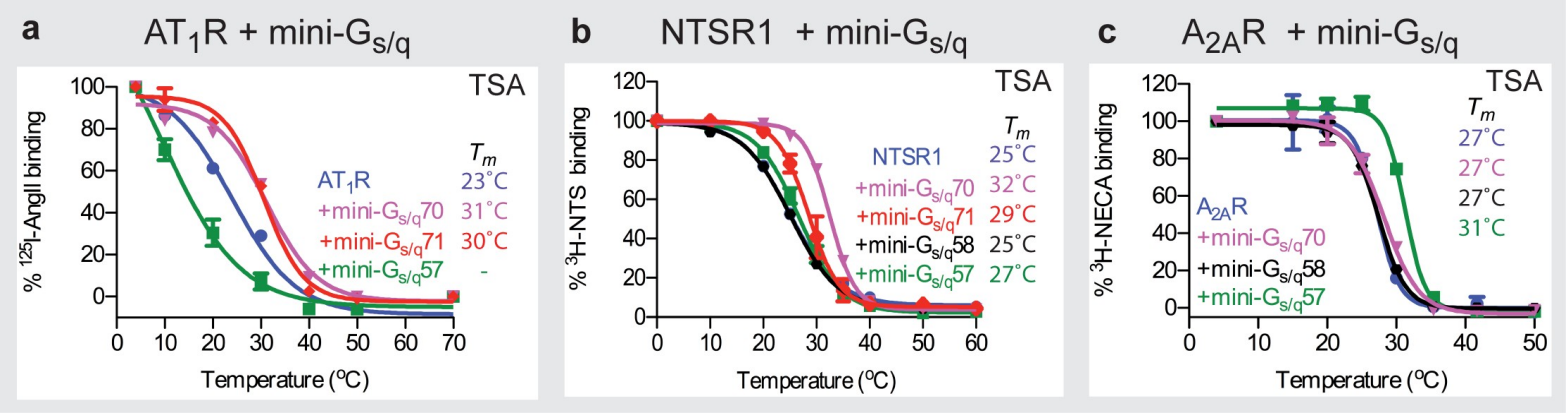
Thermostability assays of various complexes between mini-G_s/q_ chimeras and GPCRs. (**a**) Thermostability of unpurified digitonin-solubilized, ^125^I-AngII-bound AT_1_R (*T*_*m*_ values in parentheses): blue circles, no mini-G_s/q_ (23 ± 0.4°C); green squares, mini-G_s/q_57 (*T*_*m*_ not determined); magenta inverted triangles, mini-G_s/q_70 (31 ± 1°C); red triangles, mini-G_s/q_71 (30 ± 0.8°C). (**b**) Thermostability of unpurified DDM-solubilized, ^3^H-NTS-bound NTSR1: blue, no mini-G_s/q_ (25 ± 0.4°C); green squares, mini-G_s/q_57 (27 ± 0.7°C); black circles, mini-G_s/q_58 (25 ± 0.4°C); magenta inverted triangles, mini-G_s/q_70 (32 ± 0.3°C); red diamonds, mini-G_s/q_71 (29 ± 1.1°C). (**c**) Thermostability of unpurified DM-solubilized, ^3^H-NECA-bound A_2A_R: blue circles, no mini-G_s/q_ (27 ± 0.3°C); green squares, mini-G_s/q_57 (31 ± 0.3°C); black circles, mini-G_s/q_58 (27 ± 0.5°C); magenta inverted triangles, mini-G_s/q_70 (27 ± 0.2°C). In all panels, data (n = 3) were analysed by nonlinear regression and apparent *T*_*m*_ values were determined from analysis of the sigmoidal dose-response curves fitted with values shown as mean ± SEM. Curves shown are from a representative experiment.

Mini-G_s_ did not couple to any of the G_q_-coupled receptors tested (results not shown). We then evaluated a number of mini-G_s/q_ chimeras (S4 Fig) for both gain of binding to G_q_-coupled receptors (Fig 5A and 5B) and loss of binding to the cognate G_s_-coupled receptor A_2A_R, predominantly using thermostability assays (Fig 5C) and SEC (S5 Fig). First, the chimera mini-G_s/q_57 was constructed in which the five C-terminal amino acids of mini-G_s_ (Q^H5.22^YELL^H5.26^) were changed to those found in Gα_q_, which required three mutations (Q390E^H5.22^, E392N^H5.24^ and L394V^H5.26^). There was no increase in thermostability of agonist-bound AT_1_R or NTSR1 in the presence of mini-G_s/q_57 that might suggest coupling (Fig 5A and 5B). In fact, there was a decrease in thermostability of AT_1_R in the presence of mini-G_s/q_57, suggesting that binding had occurred, but that residues of G_s_ were clashing with the receptor and causing its destabilisation. Furthermore, a complex between mini-G_s/q_57 and A_2A_R was still observed (Fig 5C and S5 Fig), suggesting that the mutations were insufficient to change the specificity of G_s_ to G_q_. Therefore, the chimera mini-G_s/q_58 was constructed in which the final 19 amino acid residues in the α5 helix of mini-G_s_ (Phe376^H5.8^—Leu394^H5.26^) were changed to those in Gα_q_; this required 13 mutations (N377A^H5.9^, D378A^H5.10^, C379V^H5.11^, R380K^H5.12^, I382T^H5.14^, Q384L^H5.16^, R385Q^H5.17^, M386L^H5.18^, H387N^H5.19^, R389K^H5.21^, Q390E^H5.22^, E392N^H5.24^ and L394V^H5.26^). Mini-G_s/q_58 did not couple to A_2A_R (Fig 5C and S5 Fig), demonstrating that residues in the α5 helix beyond the C-terminal 5 amino acids are important in G protein specificity. However, there was no significant shift in the thermostability of the G_q_-coupled receptor NTSR1 in the presence of mini-G_s/q_58 (Fig 5B). We reasoned that this may be because the stability of mini-G_s/q_58 was impaired, because mutating the last 19 amino acid residues in mini-G_s_ would have also changed residues buried in the core of the G protein, thus affecting the stability of the mini-G_s_ backbone. Therefore, a refined version of this chimera, mini-G_s/q_70, was constructed in which residues in the α5 helix whose side chains formed direct contacts (3.9 Å cut-off) with either β_2_AR [2] or A_2A_R [20] in the G protein-bound structures were mutated to match those in Gα_q_ (R380K^H5.12^, Q384L^H5.16^, R385Q^H5.17^, H387N^H5.19^, E392N^H5.24^ and L394V^H5.26^; S4 Fig). In addition, the mutation Q390E^H5.22^ was included, despite only making contact to A_2A_R via its backbone, as it is buried in the receptor–G protein interface and may be important for binding to G_q_-coupled receptors. Mini-G_s/q_70 gave better binding to both G_q_-coupled receptors tested, NTSR1 and AT_1_R, and showed no binding to A_2A_R (Fig 5 and S5 Fig).

Two other chimeras were also constructed to try and improve on mini-G_s/q_70. Mini-G_s/q_72 contained the additional mutation C379V^H5.11^ compared to mini-G_s/q_70 and, although the C379^H5.11^ side chain does not form direct contacts with either A_2A_R or β_2_AR, its mutation to Val is predicted to introduce a direct interaction between the Val γ2 carbon and Leu110 from A_2A_R. However, the AT_1_R–mini-G_s/q_72 complex did not have a higher thermostability than AT_1_R–mini-G_s/q_70 (results not shown). Finally, the chimera mini-G_s/q_71 was constructed in which residues from other regions of Gα that form direct contacts with either β_2_AR [2] or A_2A_R [20] were mutated to match those in Gα_q_. This included the seven mutations in mini-G_s/q_70 (R380K^H5.12^, Q384L^H5.16^, R385Q^H5.17^, H387N^H5.19^, Q390E^H5.22^, E392N^H5.24^ and L394V^H5.26^) and six additional mutations (A39R^HNS1.3^, H41L^S1.2^, D343K^H4.23^, L346V^H4.26^, R347D^H4.27^ and Y358I ^H4S6.11^). D343^H4.23^ was the only amino acid residue whose side chain did not interact with either A_2A_R or β_2_AR, but the mutation to Lys was included because the longer side chain could potentially interact with a receptor and the charge reversal may be important for specificity. Conversely, Thr350^H4S6.3^ was *not* mutated to Pro in mini-G_s/q_71 even though its side chain forms direct contacts with β_2_AR. Alignment of Gα_s_ with two independently solved structures of Gα_q_ [47, 48] showed that this region of the G proteins differ significantly and thus, in Gα_q_, this residue is unlikely to interact with the receptor. However, after all these considerations to make an improved version of mini-G_s/q_70, mini-G_s/q_71 did not improve the thermostability of agonist-bound G_q_-coupled receptors compared to mini-G_s/q_70 (Fig 5A and 5B).

#### Mini-G_i1_: Tackling stability issues

Transfer of the 7 point mutations and 3 deletions from mini-G_s_ into Gα_i1_ to make mini-G_i1_ was not successful, as the resultant protein was very poorly expressed and had low stability (results not shown). Whilst the work on developing chimeras of mini-G_s/q_ was underway, we decided to first study the reasons why mini-G_i1_ appeared to be so unstable. Therefore, to improve expression, stability and to allow binding of the mini-G_i1_ to the βγ subunits, the N-terminus (residues 4–18) was re-inserted, Asp249^H3.8^ was mutated back to Leu, and the G217D^H2S4.3^ mutation introduced based on a sequence comparison between G_i1_ (poorly expressed) and G_s_/G_o_ (highly expressed) (Fig 2 and S1 Fig). The resultant mini-G_i1_ (construct 46) yielded only 12 mg of purified protein per litre of culture and was 17°C less stable than mini-G_s_, but was suitable for initial studies in GPCR coupling.

**Fig 6:**
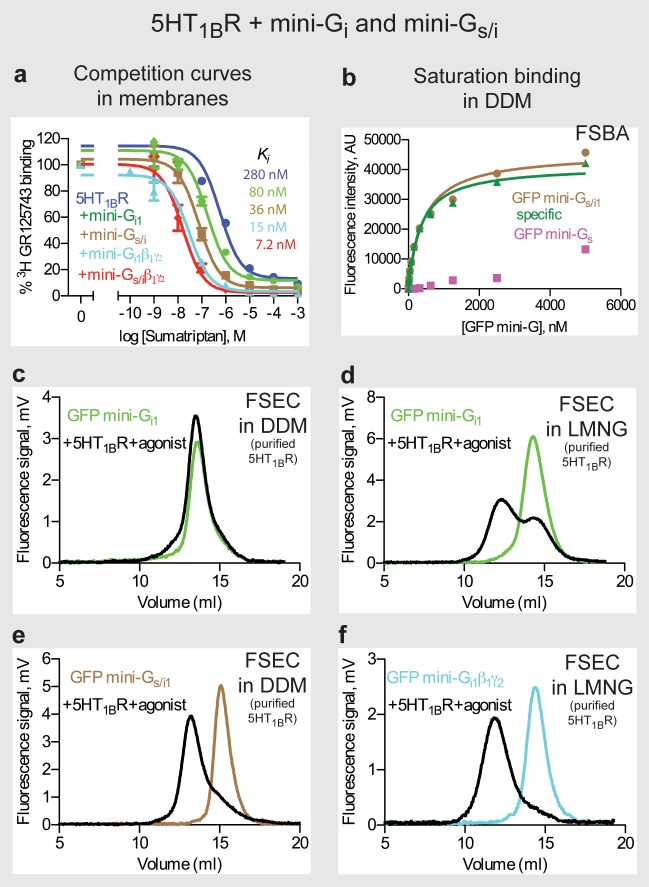
The 5HT_1B_R−mini-G_i1_ complexes. (**a**) Mini-G_i1_ coupling increases agonist affinity to 5HT_1B_R. Competition binding curves were performed in duplicate (n = 2) by measuring the displacement of the antagonist ^3^H-GR125743 with increasing concentration of the agonist sumatriptan (*K*_*i*_ values representing mean ± SEM in parentheses): blue circles, 5HT_1B_R (*K*_*i*_ 280 ± 10 nM); green hexagons, 5HT_1B_R and mini-G_i1_ (*K*_*i*_ 80 ± 13 nM); brown squares, 5HT_1B_R and mini-G_s/i1_ (*K*_*i*_ 36 ± 2 nM); pale blue triangles, 5HT_1B_R and mini-G_i1_β_1_γ_2_ (*K*_*i*_ 15 ± 1 nM); red diamonds, 5HT_1B_R and mini-G_s/i1_β_1_γ_2_ (*K*_*i*_ 7.2 ± 0.8 nM). Error bars represent the SEM. (**b**) Measurement of mini-G_s/i1_ chimera affinity to the DDM-solubilized, donitriptan-bound 5HT_1B_R using FSBA: brown circles, 5HT_1B_R and GFP-mini-G_s/i1_ (total binding); purple squares, 5HT_1B_R and GFP-mini-G_s_ (non-specific binding); green triangles, specific binding. The apparent *K*_*D*_ of 390 ± 47 nM represents the mean ± SEM of two independent experiments. Curves shown are from a representative experiment. (**c**) FSEC traces of GFP-mini-G_i1_ with 5HT_1B_R in DDM: black, GFP-mini-G_i1_ and donitriptan-bound 5HT_1B_R purified in DDM (13.5 ml); green GFP-mini-G_i1_ (13.5 ml). (**d**) FSEC traces of GFP-mini-G_i1_ with 5HT_1B_R in LMNG: black, GFP-mini-G_i1_ and donitriptan-bound 5HT_1B_R purified in LMNG (12.2 ml and 14.3 ml); green, GFP-mini-G_i1_ (14.3 ml). (**e**) FSEC traces of GFP-mini-G_s/i1_ with 5HT_1B_R: black, GFP-mini-G_s/i1_ and donitriptan-bound 5HT_1B_R purified in DDM (13.2 ml); brown, GFP-mini-G_s/i1_ (15.1 ml). (**f**) FSEC traces of GFP-mini-G_i1_β_1_γ_2_ with 5HT_1B_R: black, GFP-mini-G_i1_β_1_γ_2_ and donitriptan-bound 5HT_1B_R purified in LMNG (11.8 ml); pale blue, GFP-mini-G_i1_β_1_γ_2_ (14.3 ml). In panels **c-f**, retention volumes are given in parentheses.

The serotonin 5HT_1B_ receptor (5HT_1B_R) was used as a model G_i_-coupled receptor for developing mini-G_i1_ because it could be expressed and purified in DDM using the baculovirus expression system and its structure determined in the inactive state. Initially, GFP-mini-G_i1_ was tested using FSEC for binding to purified 5HT_1B_R (in DDM) and bound to the agonist donitriptan. However, the GFP-mini-G_i1_ (S3 Fig) migrated at 13.5 ml in the absence of receptor or in the presence of donitriptan-bound 5HT_1B_R, indicating that no coupling occurred (Fig 6C). However, when the LMNG-purified 5HT_1B_R was used, the FSEC showed two peaks, one corresponding to free GFP-mini-G_i1_ with a retention volume of 14.3 ml and the other corresponding to GFP-mini-G_i1_ bound to donitriptan-activated 5HT_1B_R, with a retention volume of 12.2 ml (Fig 6D). The presence of two peaks, suggested that mini-G_i1_ was not stable in detergent when in a complex with 5HT_1B_R. The detergent instability of the complex could also have been due to the receptor, but because agonist-bound 5HT_1B_R had been crystallised previously [10], we felt this was unlikely. In order to improve the stability of mini-G_i1_, the heterotrimer was formed between GFP-mini-G_i1_46 (S3 Fig) and β_1_γ_2_ and tested by FSEC. The GFP-mini-trimer in complex with the LMNG-purified 5HT_1B_R resolved as a single peak with a retention volume of 11.8 ml compared to 14.3 ml for the free GFP-mini-G_i1_β_1_γ_2_ trimer (Fig 6F). Thus the β_1_γ_2_ subunits restored the stability of mini-G_i1_.

Although the mini-G_i1_β_1_γ_2_ trimer coupled successfully to LMNG-solubilised 5HT_1B_R, this is not as desirable for crystallography as a mini-G protein coupled receptor due to the large size of the heterotrimeric G protein. Therefore, following the successful strategy of changing the coupling of mini-G_s_ to that of G_q_ by making a mini-G_s/q_ chimera, the same strategy was applied to engineer a mini-G_s/i1_ chimera (S1 Fig and S6 Fig). Therefore 9 mutations (C379V^H5.11^, R380T^H5.12^, Q384I^H5.16^, R385K^H5.17^, H387N^H5.19^, Q390D^H5.22^, Y391C^H5.23^, E392G^H5.24^ and L394F^H5.26^) were introduced into the α5 helix of mini-G_s_ to change its coupling specificity to that of G_i1_. A complex between GFP-mini-G_s/i1_43 (S3 Fig) with donitriptan-bound DDM-purified 5HT_1B_R resolved as a single peak with a retention volume of 13.2 ml compared to 15.1 ml for the free GFP-mini-G_s/i1_ (Fig 6E). Thus mini-G_s/i1_ was indeed more stable than mini-G_i1_. The specificity of mini-G_s_ compared to mini-G_s/i1_ for donitriptan-bound, DDM-solubilised 5HT_1B_R was confirmed using FSBA (Fig 6B). No specific coupling of GFP-mini-G_s_ to 5HT_1B_R was observed, although specific coupling to GFP-mini-G_s/i1_ (apparent *K*_*D*_ 390 nM; Fig 6B) was confirmed.

In order to compare all of the mini-G_i1_ constructs and the role of β_1_γ_2_, agonist affinity shift assays were performed on 5HT_1B_R. The uncoupled receptor showed a *K*_*i*_ for the agonist sumatriptan in this assay of 280 ± 10 nM, which was shifted by mini-G_i1_46 and mini-G_s/i1_43 to 80 ± 13 nM and 36 ± 2 nM, respectively (Fig 6A). However, addition of β_1_γ_2_ to the mini-G proteins resulted in a further increase in agonist affinity to 15 ± 1 nM and 7.2 ± 0.8 nM for mini-G_i1_46-β_1_γ_2_ and mini-G_s/i1_43-β_1_γ_2_, respectively. Thus despite the successful generation of both mini-G_i1_ and mini-G_s/i1_, their stability is still not perfect as binding of β_1_γ_2_ stabilises the mini-G proteins and elicits a greater increase in agonist affinity upon coupling of the mini-trimers.

#### Coupling of mini-G_o1_ to 5HT_1B_R

**Fig 7:**
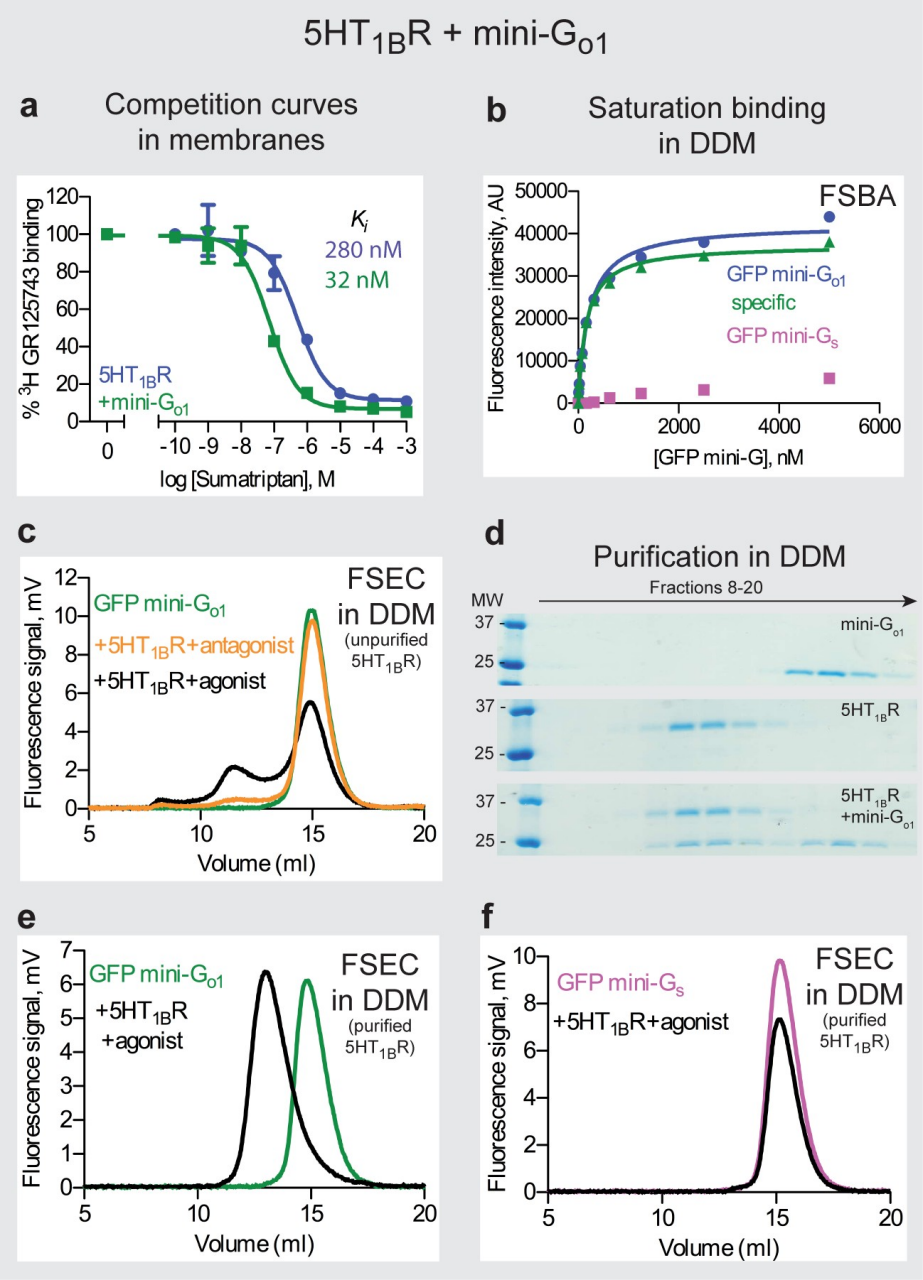
The 5HT_1B_R−mini-G_o1_ complex. (**a**) Competition binding curves were performed on membranes in duplicate (n = 2) by measuring the displacement of the antagonist ^3^H-GR125743 with increasing concentration of the agonist sumatriptan (apparent *K*_*i*_ values representing mean ± SEM are in parentheses): blue circles, 5HT_1B_R (*K*_*i*_ 280 ± 10 nM); green squares, 5HT_1B_R and mini-G_o1_ (*K*_*i*_ 32 ± 3 nM). Error bars represent SEM. (**b**) Measurement of GFP-mini-G_o1_ affinity to DDM-solubilized, donitriptan-bound 5HT_1B_R using the FSBA: blue circles, 5HT_1B_R and GFP-mini-G_o1_ (total binding); purple squares, 5HT_1B_R and GFP-mini-G_s_ (non-specific binding); green triangles, specific binding. The apparent *K*_*D*_ value (180 ± 24 nM) represents mean ± SEM of two independent experiments. Curves shown are from a representative experiment. (**c**) FSEC traces of GFP-mini-G_o1_ with DDM-solubilized unpurified 5HT_1B_R bound to the following (retention volumes are shown in parentheses): orange, the antagonist SB224289 (14.9 ml); black, the agonist donitriptan (11.3 ml and 14.9 ml). Free GFP-mini-G_o1_ (green) resolved as a predominant peak with a retention volume of 14.9 ml (**d**) Mini-G_o1_ forms a complex with purified 5HT_1B_R. The three panels are coomassie blue-stained SDS-PAGE gels of fractions from 3 separate SEC experiments: top panel, mini-G_o1_; middle panel, 5HT_1B_R; bottom panel, mini-G_o1_ mixed with donitriptan-bound 5HT_1B_R (1:1 molar ratio). (**e**) FSEC traces of GFP-mini-G_o1_ with purified 5HT_1B_R: black, GFP-mini-G_o1_ with 5HT_1B_R purified in DDM (13 ml); green, GFP-mini-G_o1_ (14.8 ml). (**f**) FSEC traces of GFP-mini-G_s_ with purified 5HT_1B_R: black, GFP-mini-G_s_ with 5HT_1B_R purified in DDM (negative control; 15.1 ml); purple, GFP-mini-G_s_ (15.1 ml). Retention volumes are shown in parentheses.

The GTPase domain of G_o1_ and G_i1_ are highly conserved (80% identity), but the mini-G proteins derived from them behaved very differently. Unlike the unstable mini-G_i1_, mini-G_o1_ expressed well (100 mg/L), had high stability comparable to mini-G_s_ and it was largely insensitive to the presence of mild detergents. Since 5HT_1B_R couples to both G_o_ and G_i_ family members [49], we tested mini-G_o1_ coupling to 5HT_1B_R and compared the results to coupling with mini-G_i1_ (see above). On FSEC, GFP-mini-G_o1_12 (S3 Fig) partially coupled to donitriptan-bound, DDM-solubilised 5HT_1B_R (unpurified), with the higher molecular weight species (retention volume 13 ml) reduced when the receptor was bound to an antagonist (Fig 7C). This was in contrast to the results with mini-G_i1_ under the same conditions where no binding was observed (Fig 6C). The partial coupling probably resulted from the low amount of 5HT_1B_R used in the assay, because when the experiment was repeated using purified 5HT_1B_R and GFP-mini-G_o1_, all of the GFP-mini-G_o1_ bound to the receptor (Fig 7E). This is consistent with what was observed for β_1_AR and A_2A_R (Fig 3). The 5HT_1B_R–mini-G_o1_ complex was purified by SEC and SDS-PAGE indicated co-elution of 5HT_1B_R and mini-G_o1_ in a 1:1 molar ratio (Fig 7D). GFP-mini-G_o1_ bound to DDM-solubilised 5HT_1B_R in the presence of donitriptan with an apparent *K*_*D*_ of 180 ± 24 nM (Fig 7B). In membranes, mini-G_o1_12 shifted the agonist affinity for 5HT_1B_R from 280 ± 10 nM to 32 ± 3 nM (Fig 7A).

The properties of mini-G_o1_ make this an ideal choice for structural studies of G_o_/G_i_ coupled receptors, rather than using mini-G_s/i_, as it is more highly expressed and more tolerant of detergents. This is exemplified by the stability of the donitriptan-bound 5HT_1B_R–mini-G_o1_ complex made from purified components (S7 Fig).

## Conclusions

The aim of the work presented here was to generate a range of mini-G proteins that could be used as a basis for the structure determination of GPCRs in their fully active state. The original work in developing mini-G proteins was performed on G_s_ [24], which turned out to be one of the best expressed and most stable of the mini-G proteins. The structures of GPCRs and G proteins are highly conserved [4], and there are thought to be highly conserved networks of side chain interactions within the GPCR [5] and G protein [6] that are essential for receptor activation and G protein activation. It therefore seemed reasonable to use the two G_s_-coupled GPCR structures [2, 20] to guide the engineering of G_i_, G_o_ or G_q_, given that there are no structures of receptors coupled to these G proteins. Transfer of the relevant mutations from mini-G_s_ to other G proteins was successful in deriving mini-G_olf_, mini-G_o1_ and mini-G_12_. Both mini-G_olf_ and mini-G_o1_ coupled to relevant receptors only in the presence of an agonist and formed stable complexes that could be purified by SEC. Currently, we have not been able to demonstrate binding of mini-G_12_ to any receptor (results not shown), even though it is highly expressed in *E*. *coli* and has high thermal stability, suggesting that the protein is in a folded state. In contrast, initial trials to generate mini-G_t1_, mini-G_z_ mini-G_q_ and mini-G_16_ were unsuccessful due to no expression in *E*. *coli*; we have not yet tested the chimera strategy on these targets. Mini-G_i1_ expressed very poorly, but was improved upon further mutagenesis, but was still not as stable as mini-G_s_ and required binding of βγ subunits to attain a full agonist affinity shift in the 5HT_1B_R.

The second approach to generate mini-G proteins for those that did not work initially was to make chimeras by converting the specificity of mini-G_s_ to the specificity of the desired G protein. This was developed initially for G_q_ by mutating in mini-G_s_ only those residues in the α5 helix whose side chains make contact to either β_2_AR or A_2A_R in the crystal structures of the relevant complexes [2, 20], to match the equivalent residues in G_q_. The final mini-G_s/q_ chimera was stable, overexpressed in *E*. *coli* and coupled to G_q_-coupled receptors, but not to G_s_-coupled receptors. The process was also successful in generating a mini-G_s/i1_ and mini-G_s/o_ chimera. The α5 helix provides ~70% of the buried surface area between the GTPase domain and the receptor in the two G protein–GPCR complexes crystallised to date. The work here shows that changing these contacts is sufficient to alter the specificity of coupling. However, this is not to say that the remaining 30% of the interface is not important, merely that a range of amino acid residues can be accommodated in this interface and therefore it plays a less important role in defining both specificity and the affinity of G protein binding.

The mini-G proteins and their properties are shown in Table 1 and Table 2. On the whole, the expression levels are satisfactory in *E*. *coli* and the stability of the mini-G proteins in the absence of detergent is also good. However, their stability decreases in detergent, particularly in high concentrations, with the greatest decrease in stability observed at high detergent concentrations (greater than 0.5% w/v) and with detergents that are regarded as harsh for membrane protein purification [50]. Thus care must be exercised in the initial choice of detergent for forming receptor–mini-G protein complexes. However, GPCRs in the active state are also unstable in detergent and this also needs to be carefully addressed before structural studies are a possibility. This may require the use of very high-affinity agonists that stabilize the receptor and/or the stabilisation of the GPCR–mini-G protein complex by conformational thermostabilisation. It is not trivial to define which component, receptor or mini-G protein, is the least stable within a complex under a given set of conditions. This is why we recommend that initial tests are performed on the receptor solubilized in very mild detergents such as digitonin, GDN or LMNG.

**Table 1:** Mini-G proteins and their characteristics.

Mini-G protein	Construct	Yield of pure protein per L of *E*. *coli* (mg)	Stability measured by DSF (°C)	Stability in detergent measured by native DSF (°C)		
				No detergent	0.1% LMNG	0.1% DDM
G_s_	393	100	65 ± 0.0	48 ± 0.2	45 ± 0.2	39 ±0.0
G_olf_	6	80	65 ± 0.4	44 ± 0.1	42 ± 0.0	37 ± 0.2
G_s/q_	70	50	67 ± 0.4	47 ± 0.3	44 ± 0.2	36 ±0.1
G_s/i1_	43	40	69 ± 0.1	45 ± 0.0	41 ± 0.1	36 ± 0.1
G_o1_	12	100	64 ± 0.1	44 ± 0.2	41 ± 0.1	33 ±0.2
G_12_	8	25	73 ± 0.3	50 ± 0.1	46 ± 0.1	41 ± 0.2

**Table 2:** Mini-G proteins that bind βγ subunits and their characteristics.

Mini-G protein that bind βγ	Construct	Yield of pure protein per L of *E*. *coli* (mg)	Stability measured by DSF (°C)
G_s_	399	100	72 ± 0.0
G_olf_	9	144	66 ± 0.1
G_s/q_	76	30	71 ± 0.1
G_i1_	46	12	48 ± 0.3
G_s/i1_	48	10	72 ± 0.1
G_s/o1_	16	15	69 ± 0.1

Despite the successes described here, mini-G proteins are not a panacea for all GPCRs. The specificity determinants for G protein coupling are not determined solely by the α5 helix and the subtypes of βγ can also affect the efficiency and specificity of coupling within a cell [51, 52]. As we have not taken any subtype-specific G protein-GPCR interactions into account, further engineering of the mini-G proteins in the light of new structural data of GPCRs coupled to G_i_, G_o_ or G_q_ may improve their performance further. Additional complications to making stable receptor–G protein complexes are the kinetics of dissociation of the G protein from the activated receptor and the stability of the activated receptor itself. There has probably been strong evolutionary pressure for some GPCRs to evolve transient signaling complexes that are inherently unstable to provide tight regulation of the signaling process. Thus it may be necessary to thermostabilise the receptor in the active conformation [36] to generate a complex sufficiently stable for structural studies, although for other GPCR–G protein complexes this may not be necessary.

## Supporting information

S1 Fig Sequence of mini-G proteins used in this study. The poly-histidine tag is highlighted in red, the TEV protease cleavage site is highlighted in grey, and the linker used to replace the GαAH domain is highlighted in turquoise. Mutations are shown in bold type and underlined.
(DOCX)

S2 Fig Sequence of mini-G proteins that were *not* successfully expressed in *E*. *coli*. The poly-histidine tag is highlighted in red, the TEV cleavage site highlighted in grey and the linker used to replace the GαAH domain is highlighted in turquoise. Mutations are shown in bold type and underlined. The constructs were cloned into plasmid pET15b for *E*. *coli* expression using *Nco*I (yellow) and *Xho*I (magenta) restriction sites. Start and stop codons are in red.
(DOCX)

S3 Fig Sequence of GFP-mini-G proteins used in this study. GFP (highlighted in green) was fused to the N-terminus of the mini-G proteins with a GGGGS linker (highlighted in yellow). The poly-histidine tag is highlighted in red, the TEV cleavage site highlighted in grey and the linker used to replace the GαAH domain is highlighted in turquoise.
(DOCX)

S4 Fig Sequence alignment of selected mini-G_s/q_ chimeras used in this study. Residues in red are the signature mutations of a mini-G protein. Residues in blue are those found in G_q_. Diamonds above the sequences identify the amino acid residues in Gα_s_ where the side chains that make atomic contacts to residues in either β_2_AR (β2 con) or A_2A_R (2A con). Ovals above the sequences identify the amino acid residues in Gα_s_ where only the main chain atoms make contacts to the receptor.
(DOCX)

S5 Fig Analytical SEC and SDS-PAGE analyses of purified A_2A_R with mini-G_s/q_ chimeras. Analytical SEC of mini-G_s/q_57 (**a**), mini-G_s/q_58 (**b**) and mini-G_s/q_70 (**c**) bound to purified A_2A_R: black, A_2A_R–mini-G_s/q_ complex; blue, A_2A_R; green, mini-G_s/q_. Three panels below the SEC traces are coomassie blue-stained SDS-PAGE gels of fractions from 3 separate SEC experiments: top panel, mini-G_s/q_; middle panel, A_2A_R; bottom panel, NECA-bound A_2A_R mixed with mini-G_s/q_ (1:1.2 molar ratio).
(PDF)

S6 Fig Sequence alignment of the different mini-G_i1_ and mini-G_o1_ proteins used in this study. Residues in red are the signature mutations of a mini-G protein. Note the additional G217D mutation (highlighted in yellow; residue 114 in the mini-G protein) in mini-G_i1_ to improve expression. Residues highlighted in cyan in mini-G_s_ were mutated to their equivalent in mini-G_i1_ or mini-G_o1_ (highlighted in magenta and grey respectively) to make the mini-G_s/i1_ or mini-G_s/o1_ chimeras. Note the re-insertion of the N-terminus and the back mutation (D to L) highlighted in green in the constructs that were used to form a heterotrimer with β_1_γ_2_ (*i*.*e*. mini-G_i1__46; mini-G_s/i1__43 and mini-G_s/o1__16).
(PDF)

S7 Fig Stability of the purified donitriptan-bound 5HT_1B_R–mini-G_o1_ complex. (a) The complex was assembled from purified components and analysed by SEC immediately after assembly (blue line) or after the sample was stored at 4°C for 48h (red line). Fractions (0.5 ml) were collected and analysed by SDS-PAGE: (b) immediately after assembly; (c) after 48 h at 4°C.
(PDF)
