# Supplementary material for: Mini-G proteins: Novel tools for studying GPCRs in their active conformation
Source: PLoS One. 2017 Apr 20;12(4):e0175642. doi: 10.1371/journal.pone.0175642 (PMC5398546; doi:10.1371/journal.pone.0175642)
Supplement: S1 Fig — The poly-histidine tag is highlighted in red, the TEV protease cleavage site is highlighted in grey, and the linker used to replace the GαAH domain is highlighted in turquoise. Mutations are shown in bold type and underlined. (DOCX) [file pone.0175642.s001.docx]

>Mini-G_s_393

MGHHHHHHENLYFQGIEKQLQKDKQVYRATHRLLLLGA**DN**SGKSTIVKQMRILHGGSGGSGGTSGIFETKFQVDKVNFHMFDVGGQRDERRKWIQCFNDVTAIIFVV**D**SS**D**YNRLQEALN**D**FKSIWNNRWLRTISVILFLNKQDLLAEKVLAGKSKIEDYFPEFARYTTPEDATPEPGEDPRVTRAKYFIRDEFLRISTASGDGRHYCYPHFTCAVDTEN**A**RR**I**FNDCRDIIQRMHLRQYELL

>Mini-G_olf_6

MGHHHHHHENLYFQGIEKQLQKERLAYKATHRLLLLGA**DN**SGKSTIVKQMRILHGGSGGSGGTSGIFETRFQVDKVNFHMFDVGGQRDERRKWIQCFNDVTAIIYVA**D**CS**D**YNRLRESLD**D**FESIWNNRWLRTISIILFLNKQDMLAEKVLAGKSKIEDYFPEYANYTVPEDATPDAGEDPKVTRAKFFIRDLFLRISTATGDGKHYCYPHFTCAVDTEN**A**RR**I**FNDCRDIIQRMHLKQYELL

>Mini-G_s/q_57

MGHHHHHHENLYFQGIEKQLQKDKQVYRATHRLLLLGA**DN**SGKSTIVKQMRILHGGSGGSGGTSGIFETKFQVDKVNFHMFDVGGQRDERRKWIQCFNDVTAIIFVV**D**SS**D**YNRLQEALN**D**FKSIWNNRWLRTISVILFLNKQDLLAEKVLAGKSKIEDYFPEFARYTTPEDATPEPGEDPRVTRAKYFIRDEFLRISTASGDGRHYCYPHFTCAVDTEN**A**RR**I**FNDCRDIIQRMHLR**E**Y**N**L**V**

>Mini-G_s/q_58

MGHHHHHHENLYFQGIEKQLQKDKQVYRATHRLLLLGA**DN**SGKSTIVKQMRILHGGSGGSGGTSGIFETKFQVDKVNFHMFDVGGQRDERRKWIQCFNDVTAIIFVV**D**SS**D**YNRLQEALN**D**FKSIWNNRWLRTISVILFLNKQDLLAEKVLAGKSKIEDYFPEFARYTTPEDATPEPGEDPRVTRAKYFIRDEFLRISTASGDGRHYCYPHFTCAVDTEN**A**RR**I**F**AAVK**D**T**I**LQLN**L**KE**Y**N**L**V**

>Mini-G_s/q_70

MGHHHHHHENLYFQGIEKQLQKDKQVYRATHRLLLLGA**DN**SGKSTIVKQMRILHGGSGGSGGTSGIFETKFQVDKVNFHMFDVGGQRDERRKWIQCFNDVTAIIFVV**D**SS**D**YNRLQEALN**D**FKSIWNNRWLRTISVILFLNKQDLLAEKVLAGKSKIEDYFPEFARYTTPEDATPEPGEDPRVTRAKYFIRDEFLRISTASGDGRHYCYPHFTCAVDTEN**A**RR**I**FNDC**K**DII**LQ**M**N**LR**E**Y**N**L**V**

>Mini-G_s/q_71

MGHHHHHHENLYFQGIEKQLQKDKQVYR**R**T**L**RLLLLGA**DN**SGKSTIVKQMRILHGGSGGSGGTSGIFETKFQVDKVNFHMFDVGGQRDERRKWIQCFNDVTAIIFVV**D**SS**D**YNRLQEALN**D**FKSIWNNRWLRTISVILFLNKQDLLAEKVLAGKSKIEDYFPEFARYTTPEDATPEPGEDPRVTRAKYFIR**K**EF**VD**ISTASGDGRH**I**CYPHFTCAVDTEN**A**RR**I**FNDC**K**DII**LQ**M**N**LR**E**Y**N**L**V**

>Mini-G_i1_46

MGHHHHHHENLYFQGTLSAEDKAAVERSKMIDRNLREDGEKAAREVKLLLLGA**DN**SGKSTIVKQMKIIHGGGGGGGGTTGIVETHFTFKDLHFKMFDVGGQRSERKKWIHCFE**D**VAAIIFCV**D**LSDYNRMHESMKLFDSICNNKWFTDTSIILFLNKKDLFEEKIKKSPLTICYQEYAGSNTYEEAAAYIQCQFEDLNKRKDTKEIYTHFTCATDTKN**A**QF**I**FDAVTDVIIKNNLKDCGLF

>Mini-G_s/i1_43

MGHHHHHHENLYFQGIEKQLQKDKQVYRATHRLLLLGA**DN**SGKSTIVKQMRILHGGSGGSGGTSGIFETKFQVDKVNFHMFDVGGQRDERRKWIQCFNDVTAIIFVV**D**SS**D**YNRLQEALN**D**FKSIWNNRWLRTISVILFLNKQDLLAEKVLAGKSKIEDYFPEFARYTTPEDATPEPGEDPRVTRAKYFIRDEFLRISTASGDGRHYCYPHFTCAVDTEN**A**RR**I**FND**VT**DII**IK**M**N**LR**DCG**L**F**

>Mini-G_s/i1_48

MGHHHHHHENLYFQGNSKTEDQRNEEKAQREANKKIEKQLQKDKQVYRATHRLLLLGA**DN**SGKSTIVKQMRILHGGSGGSGGTSGIFETKFQVDKVNFHMFDVGGQRDERRKWIQCFNDVTAIIFVV**D**SS**D**YNRLQEALNLFKSIWNNRWLRTISVILFLNKQDLLAEKVLAGKSKIEDYFPEFARYTTPEDATPEPGEDPRVTRAKYFIRDEFLRISTASGDGRHYCYPHFTCAVDTEN**A**RR**I**FND**VT**DII**IK**M**N**LR**DCG**L**F**

>Mini-G_o1_12

MGHHHHHHENLYFQGIEKNLKEDGISAAKDVKLLLLGA**DN**SGKSTIVKQMKIIHGGSGGSGGTTGIVETHFTFKNLHFRLFDVGGQRSERKKWIHCFEDVTAIIFCV**D**LS**D**YNRMHESLMDFDSICNNKFFIDTSIILFLNKKDLFGEKIKKSPLTICFPEYTGPNTYEDAAAYIQAQFESKNRSPNKEIYCHMTCATDTNN**A**QV**I**FDAVTDIIIANNLRGCGLY

>Mini-G_12_8

MGHHHHHHENLYFQGIDALLARERRAVRRLVKILLLGA**DN**SGKSTFLKQMRIIHGGSGGSGGTKGIVEHDFVIKKIPFKMVDVGGQRSQRQKWFQCFDGITSILFMV**D**SS**D**YNRLVESMN**D**FETIVNNKLFFNVSIILFLNKMDLLVEKVKTVSIKKHFPDFRGDPHRLEDVQRYLVQCFDRKRRNRSKPLFHHFTTAIDTEN**A**RF**I**FHAVKDTILQENLKDIMLQ

**S1 Fig.** **Sequence of mini-G proteins used in this study.**
