# Supplementary material for: Mini-G proteins: Novel tools for studying GPCRs in their active conformation
Source: PLoS One. 2017 Apr 20;12(4):e0175642. doi: 10.1371/journal.pone.0175642 (PMC5398546; doi:10.1371/journal.pone.0175642)
Supplement: S2 Fig — The poly-histidine tag is highlighted in red, the TEV cleavage site highlighted in grey and the linker used to replace the GαAH domain is highlighted in turquoise. Mutations are shown in bold type and underlined. The constructs were cloned into plasmid pET15b for E. coli expression using NcoI (yellow) and XhoI (magenta) restriction sites. Start and stop codons are in red. (DOCX) [file pone.0175642.s002.docx]

**>Mini-G_t1_**

CCATGGGTCACCACCATCACCATCATGAAAATCTTTATTTCCAGGGTCTGGAAAAGAAGCTGAAAGAGGACGCTGAGAAGGATGCTCGAACCGTGAAGCTGCTGCTTCTGGGTGCCG**A**T**A**A**T**TCCGGGAAGAGCACCATCGTCAAGCAGATGAAGATTATCCACGGTGGGAGTGGCGGGAGCGGAGGTACCACTGGCATCATCGAGACGCAGTTCTCCTTCAAGGATCTCAACTTCCGGATGTTCGATGTGGGCGGGCAGCGCTCGGAGCGCAAGAAGTGGATCCACTGCTTCGAGGGCGTGACCTGCATCATCTTCATCGCGG**AC**CTGAGCG**AT**TACAACCGCATGCACGAGAGCCTGCAC**GAT**TTCAACAGCATCTGCAACCACCGCTACTTCGCCACGACGTCCATCGTGCTCTTCCTTAACAAGAAGGACGTCTTCTTCGAGAAGATCAAGAAGGCGCACCTCAGCATCTGTTTCCCGGACTACGATGGACCCAACACCTACGAGGACGCCGGCAACTACATCAAGGTGCAGTTCCTCGAGCTCAACATGCGGCGCGACGTGAAGGAGATCTATTCCCACATGACGTGCGCCACCGACACGCAGAACG**C**CAAATTT**A**TCTTCGACGCTGTCACCGACATCATCATCAAGGAGAACCTCAAAGACTGTGGCCTCTTCTAATAGCTCGAG

MGHHHHHHENLYFQGLEKKLKEDAEKDARTVKLLLLGA**DN**SGKSTIVKQMKIIHGGSGGSGGTTGIIETQFSFKDLNFRMFDVGGQRSERKKWIHCFEGVTCIIFIA**D**LS**D**YNRMHESLH**D**FNSICNHRYFATTSIVLFLNKKDVFFEKIKKAHLSICFPDYDGPNTYEDAGNYIKVQFLELNMRRDVKEIYSHMTCATDTQN**A**KF**I**FDAVTDIIIKENLKDCGLF

**>Mini-G_z_**

CCATGGGTCACCACCATCACCATCATATTGACCGCCACCTGCGCTCAGAGAGCCAGCGGCAACGCCGCGAAATCAAGCTGCTCCTGCTGGGCACC**GAC**AACTCAGGCAAGAGCACCATCGTCAAACAGATGAAGATCATCCACGGAGGGGGCGGAGGCGGGGGAGGGACCACGGGCATTGTGGAGAACAAGTTCACCTTCAAGGAGCTCACCTTCAAGATGGTGGACGTGGGGGGGCAGAGGTCAGAGCGCAAAAAGTGGATCCACTGCTTCGAGGGCGTCACAGCCATCATCTTCTGTGTG**GAC**CTCAGC**GAC**TACAGTCGGATGGCAGAGAGCTTGCGC**GAC**TTTGACTCCATCTGCAACAACAACTGGTTCATCAACACCTCACTCATCCTCTTCCTGAACAAGAAGGACCTGCTGGCAGAGAAGATCCGCCGCATCCCGCTCACCATCTGCTTTCCCGAGTACAAGGGCCAGAACACGTACGAGGAGGCCGCTGTCTACATCCAGCGGCAGTTTGAAGACCTGAACCGCAACAAGGAGACCAAGGAGATCTACTCCCACTTCACCTGCGCCACCGACACCAGTAAC**GCG**CAGTTT**ATC**TTCGACGCGGTGACAGACGTCATCATACAGAACAATCTCAAGTACATTGGCCTTTGCTGATAACTCGAG

MGHHHHHHIDRHLRSESQRQRREIKLLLLGT**D**NSGKSTIVKQMKIIHGGGGGGGGTTGIVENKFTFKELTFKMVDVGGQRSERKKWIHCFEGVTAIIFCV**D**LS**D**YSRMAESLR**D**FDSICNNNWFINTSLILFLNKKDLLAEKIRRIPLTICFPEYKGQNTYEEAAVYIQRQFEDLNRNKETKEIYSHFTCATDTSN**A**QF**I**FDAVTDVIIQNNLKYIGLC

**>Mini-G_q_**

CCATGGGTCACCACCATCACCATCATATCGAGCGGCAGCTCCGCAGGGACAAGCGGGACGCCCGCCGGGAGCTCAAGCTGCTGCTGCTCGGGACA**GACAAC**AGTGGCAAGAGTACGTTTATCAAGCAGATGAGAATCATCCACGGAGGGGGCGGAGGCGGGGGAGGGACCACAGGGATCATCGAATACCCCTTTGACTTACAAAGTGTCATTTTCAGAATGGTCGATGTAGGGGGCCAAAGGTCAGAGAGAAGAAAATGGATACACTGCTTTGAAAATGTCACCTCTATCATGTTTCTAGTA**GAC**CTTAGT**GAC**TATAACCGAATGGAGGAAAGCAAGGCT**GAC**TTTAGAACAATTATCACATACCCCTGGTTCCAGAACTCCTCGGTTATTCTGTTCTTAAACAAGAAAGATCTTCTAGAGGAGAAAATCATGTATTCCCATCTAGTCGACTACTTCCCAGAATATGATGGACCCCAGAGAGATGCCCAGGCAGCCCGAGAATTCATTCTGAAGATGTTCGTGGACCTGAACCCAGACAGTGACAAAATTATCTACTCCCACTTCACGTGCGCCACAGACACCGAGAAT**GCC**CGCTTT**ATC**TTTGCTGCCGTCAAGGACACCATCCTCCAGTTGAACCTGAAGGAGTACAATCTGGTCTAATAGCTCGAG

MGHHHHHHIERQLRRDKRDARRELKLLLLGT**DN**SGKSTFIKQMRIIHGGGGGGGGTTGIIEYPFDLQSVIFRMVDVGGQRSERRKWIHCFENVTSIMFLV**D**LS**D**YNRMEESKA**D**FRTIITYPWFQNSSVILFLNKKDLLEEKIMYSHLVDYFPEYDGPQRDAQAAREFILKMFVDLNPDSDKIIYSHFTCATDTEN**A**RF**I**FAAVKDTILQLNLKEYNLV

**>Mini-G_16_**

CCATGGGTCACCACCATCACCATCATATCAACAGGATCCTCTTGGAGCAGAAGAAGCAGGACCGCGGGGAGCTGAAGCTGCTGCTTTTGGGCCCA**GACAAC**AGCGGGAAGAGCACCTTCATCAAGCAGATGCGGATCATCCACGGAGGGGGCGGAGGCGGGGGAGGGACCACTGGCATCAACGAGTACTGCTTCTCCGTGCAGAAAACCAACCTGCGGATCGTGGACGTCGGGGGCCAGAAGTCAGAGCGTAAGAAATGGATCCATTGTTTCGAGAACGTGATCGCCCTCATCTACCTGGCC**GAC**CTGAGT**GAC**TACAACCGCATGAAGGAGAGCCTCGCA**GAC**TTTGGGACTATCCTGGAACTACCCTGGTTCAAAAGCACATCCGTCATCCTCTTTCTCAACAAAACCGACATCCTGGAGGAGAAAATCCCCACCTCCCACCTGGCTACCTATTTCCCCAGTTTCCAGGGCCCTAAGCAGGATGCTGAGGCAGCCAAGAGGTTCATCCTGGACATGTACACGAGGATGTACACCGGGTGCGTGGACGGCCCCGAGGGCAGCAAGAAGGGCGCACGATCCCGACGCCTCTTCAGCCACTACACATGTGCCACAGACACACAGAAC**GCG**CGCAAG**ATC**TTCAAGGACGTGCGGGACTCGGTGCTCGCCCGCTACCTGGACGAGATCAACCTGCTGTAATAGCTCGAG

MGHHHHHHINRILLEQKKQDRGELKLLLLGP**DN**SGKSTFIKQMRIIHGGGGGGGGTTGINEYCFSVQKTNLRIVDVGGQKSERKKWIHCFENVIALIYLA**D**LS**D**YNRMKESLA**D**FGTILELPWFKSTSVILFLNKTDILEEKIPTSHLATYFPSFQGPKQDAEAAKRFILDMYTRMYTGCVDGPEGSKKGARSRRLFSHYTCATDTQN**A**RK**I**FKDVRDSVLARYLDEINLL

**S2 Fig. Sequence of mini-G proteins that were *not* successfully expressed in *E. coli*.**
