# Supplementary material for: Mini-G proteins: Novel tools for studying GPCRs in their active conformation
Source: PLoS One. 2017 Apr 20;12(4):e0175642. doi: 10.1371/journal.pone.0175642 (PMC5398546; doi:10.1371/journal.pone.0175642)
Supplement: S3 Fig — GFP (highlighted in green) was fused to the N-terminus of the mini-G proteins with a GGGGS linker (highlighted in yellow). The poly-histidine tag is highlighted in red, the TEV cleavage site highlighted in grey and the linker used to replace the GαAH domain is highlighted in turquoise. (DOCX) [file pone.0175642.s003.docx]

>GFP-mini-G_s_393

MGHHHHHHENLYFQGVSKGEELFTGVVPILVELDGDVNGHKFSVSGEGEGDATYGKLTLKFICTTGKLPVPWPTLVTTLTYGVQCFSRYPDHMKQHDFFKSAMPEGYVQERTIFFKDDGNYKTRAEVKFEGDTLVNRIELKGIDFKEDGNILGHKLEYNYNSHNVYIMADKQKNGIKVNFKIRHNIEDGSVQLADHYQQNTPIGDGPVLLPDNHYLSTQSALSKDPNEKRDHMVLLEFVTAAGITLGMDELYKGGGGSIEKQLQKDKQVYRATHRLLLLGA**DN**SGKSTIVKQMRILHGGSGGSGGTSGIFETKFQVDKVNFHMFDVGGQRDERRKWIQCFNDVTAIIFVV**D**SS**D**YNRLQEALN**D**FKSIWNNRWLRTISVILFLNKQDLLAEKVLAGKSKIEDYFPEFARYTTPEDATPEPGEDPRVTRAKYFIRDEFLRISTASGDGRHYCYPHFTCAVDTEN**A**RR**I**FNDCRDIIQRMHLRQYELL

>GFP-mini-G_i1_46

MGHHHHHHENLYFQGVSKGEELFTGVVPILVELDGDVNGHKFSVSGEGEGDATYGKLTLKFICTTGKLPVPWPTLVTTLTYGVQCFSRYPDHMKQHDFFKSAMPEGYVQERTIFFKDDGNYKTRAEVKFEGDTLVNRIELKGIDFKEDGNILGHKLEYNYNSHNVYIMADKQKNGIKVNFKIRHNIEDGSVQLADHYQQNTPIGDGPVLLPDNHYLSTQSALSKDPNEKRDHMVLLEFVTAAGITLGMDELYKGGGGSTLSAEDKAAVERSKMIDRNLREDGEKAAREVKLLLLGA**DN**SGKSTIVKQMKIIHGGGGGGGGTTGIVETHFTFKDLHFKMFDVGGQRSERKKWIHCFE**D**VAAIIFCV**D**LSDYNRMHESMKLFDSICNNKWFTDTSIILFLNKKDLFEEKIKKSPLTICYQEYAGSNTYEEAAAYIQCQFEDLNKRKDTKEIYTHFTCATDTKN**A**QF**I**FDAVTDVIIKNNLKDCGLF

>GFP-mini-G_s/i1_43

MGHHHHHHENLYFQGVSKGEELFTGVVPILVELDGDVNGHKFSVSGEGEGDATYGKLTLKFICTTGKLPVPWPTLVTTLTYGVQCFSRYPDHMKQHDFFKSAMPEGYVQERTIFFKDDGNYKTRAEVKFEGDTLVNRIELKGIDFKEDGNILGHKLEYNYNSHNVYIMADKQKNGIKVNFKIRHNIEDGSVQLADHYQQNTPIGDGPVLLPDNHYLSTQSALSKDPNEKRDHMVLLEFVTAAGITLGMDELYKGGGGSIEKQLQKDKQVYRATHRLLLLGA**DN**SGKSTIVKQMRILHGGSGGSGGTSGIFETKFQVDKVNFHMFDVGGQRDERRKWIQCFNDVTAIIFVV**D**SS**D**YNRLQEALN**D**FKSIWNNRWLRTISVILFLNKQDLLAEKVLAGKSKIEDYFPEFARYTTPEDATPEPGEDPRVTRAKYFIRDEFLRISTASGDGRHYCYPHFTCAVDTEN**A**RR**I**FND**VT**DII**IK**M**N**LR**DCG**L**F**

>GFP-mini-G_o1_12

MGHHHHHHENLYFQGVSKGEELFTGVVPILVELDGDVNGHKFSVSGEGEGDATYGKLTLKFICTTGKLPVPWPTLVTTLTYGVQCFSRYPDHMKQHDFFKSAMPEGYVQERTIFFKDDGNYKTRAEVKFEGDTLVNRIELKGIDFKEDGNILGHKLEYNYNSHNVYIMADKQKNGIKVNFKIRHNIEDGSVQLADHYQQNTPIGDGPVLLPDNHYLSTQSALSKDPNEKRDHMVLLEFVTAAGITLGMDELYKGGGGSIEKNLKEDGISAAKDVKLLLLGA**DN**SGKSTIVKQMKIIHGGSGGSGGTTGIVETHFTFKNLHFRLFDVGGQRSERKKWIHCFEDVTAIIFCV**D**LS**D**YNRMHESLMDFDSICNNKFFIDTSIILFLNKKDLFGEKIKKSPLTICFPEYTGPNTYEDAAAYIQAQFESKNRSPNKEIYCHMTCATDTNN**A**QV**I**FDAVTDIIIANNLRGCGLY

>GFP-mini-G_12_8

MGHHHHHHENLYFQGVSKGEELFTGVVPILVELDGDVNGHKFSVSGEGEGDATYGKLTLKFICTTGKLPVPWPTLVTTLTYGVQCFSRYPDHMKQHDFFKSAMPEGYVQERTIFFKDDGNYKTRAEVKFEGDTLVNRIELKGIDFKEDGNILGHKLEYNYNSHNVYIMADKQKNGIKVNFKIRHNIEDGSVQLADHYQQNTPIGDGPVLLPDNHYLSTQSALSKDPNEKRDHMVLLEFVTAAGITLGMDELYKGGGGSIDALLARERRAVRRLVKILLLGA**DN**SGKSTFLKQMRIIHGGSGGSGGTKGIVEHDFVIKKIPFKMVDVGGQRSQRQKWFQCFDGITSILFMV**D**SS**D**YNRLVESMN**D**FETIVNNKLFFNVSIILFLNKMDLLVEKVKTVSIKKHFPDFRGDPHRLEDVQRYLVQCFDRKRRNRSKPLFHHFTTAIDTEN**A**RF**I**FHAVKDTILQENLKDIMLQ

**S3 Fig.** **Sequence of GFP-mini-G proteins used in this study**.
