# Supplementary material for: Mini-G proteins: Novel tools for studying GPCRs in their active conformation
Source: PLoS One. 2017 Apr 20;12(4):e0175642. doi: 10.1371/journal.pone.0175642 (PMC5398546; doi:10.1371/journal.pone.0175642)
Supplement: S4 Fig — Residues in red are the signature mutations of a mini-G protein. Residues in blue are those found in Gq. Diamonds above the sequences identify the amino acid residues in Gαs where the side chains that make atomic contacts to residues in either β2AR (β2 con) or A2AR (2A con). Ovals above the sequences identify the amino acid residues in Gαs where only the main chain atoms make contacts to the receptor. (DOCX) [file pone.0175642.s004.docx]

2A con ◊

β2 con 0◊ ◊

mini-Gq 1 MGHHHHHHENLYFQGIERQLRRDKRDARRELKLLLLGTDNSGKSTFIKQMRIIHGGGGGG 60

mini-Gs 1 MGHHHHHHENLYFQGIEKQLQKDKQVYRATHRLLLLGADNSGKSTIVKQMRILHGGSGGS 60

mini-Gs/q57 1 MGHHHHHHENLYFQGIEKQLQKDKQVYRATHRLLLLGADNSGKSTIVKQMRILHGGSGGS 60

mini-Gs/q58 1 MGHHHHHHENLYFQGIEKQLQKDKQVYRATHRLLLLGADNSGKSTIVKQMRILHGGSGGS 60

mini-Gs/q70 1 MGHHHHHHENLYFQGIEKQLQKDKQVYRATHRLLLLGADNSGKSTIVKQMRILHGGSGGS 60

mini-Gs/q71 1 MGHHHHHHENLYFQGIEKQLQKDKQVYRRTLRLLLLGADNSGKSTIVKQMRILHGGSGGS 60

*****************.**..**. * .*****.******* .*****.*** **

34567890123 1234567 1234567

HN4 5 | S1 | H1 |

2A con 0 ◊

β2 con ◊

mini-Gq 61 GGTTGIIEYPFDLQSVIFRMVDVGGQRSERRKWIHCFENVTSIMFLVDLSDYNRMEESKA 120

mini-Gs 61 GGTSGIFETKFQVDKVNFHMFDVGGQRDERRKWIQCFNDVTAIIFVVDSSDYNRLQEALN 120

mini-Gs/q57 61 GGTSGIFETKFQVDKVNFHMFDVGGQRDERRKWIQCFNDVTAIIFVVDSSDYNRLQEALN 120

mini-Gs/q58 61 GGTSGIFETKFQVDKVNFHMFDVGGQRDERRKWIQCFNDVTAIIFVVDSSDYNRLQEALN 120

mini-Gs/q70 61 GGTSGIFETKFQVDKVNFHMFDVGGQRDERRKWIQCFNDVTAIIFVVDSSDYNRLQEALN 120

mini-Gs/q71 61 GGTSGIFETKFQVDKVNFHMFDVGGQRDERRKWIQCFNDVTAIIFVVDSSDYNRLQEALN 120

***.** * * . * *.* ****** ******.** **.*.*.** *****..*.

12345678 12345678 1234567890 1234567 1234567

S2 | S3 | H2 1 S4 | H3

2A con

β2 con ◊

mini-Gq 121 DFRTIITYPWFQNSSVILFLNKKDLLEEKIMY--SHLVDYFPEYD------------GPQ 166

mini-Gs 121 DFKSIWNNRWLRTISVILFLNKQDLLAEKVLAGKSKIEDYFPEFARYTTPEDATPEPGED 180

mini-Gs/q57 121 DFKSIWNNRWLRTISVILFLNKQDLLAEKVLAGKSKIEDYFPEFARYTTPEDATPEPGED 180

mini-Gs/q58 121 DFKSIWNNRWLRTISVILFLNKQDLLAEKVLAGKSKIEDYFPEFARYTTPEDATPEPGED 180

mini-Gs/q70 121 DFKSIWNNRWLRTISVILFLNKQDLLAEKVLAGKSKIEDYFPEFARYTTPEDATPEPGED 180

mini-Gs/q71 121 DFKSIWNNRWLRTISVILFLNKQDLLAEKVLAGKSKIEDYFPEFARYTTPEDATPEPGED 180

**..* . * .. ********.*** **.. *.. *****. *

89012345678 1234567 12345678901234567 12345678901

1 | S5 | HG 1 | H4 1

2A con ◊ 0◊◊ ◊◊◊ ◊◊ 0◊

β2 con 0 ◊◊ ◊ ◊ ◊ ◊◊ ◊◊◊ ◊◊ ◊

mini-Gq 167 RDAQAAREFILKMFVDLNPDSD---KIIYSHFTCATDTENARFIFAAVKDTILQLNLKEY 223

mini-Gs 181 PRVTRAKYFIRDEFLRISTASGDGRHYCYPHFTCAVDTENARRIFNDCRDIIQRMHLRQY 240

mini-Gs/q57 181 PRVTRAKYFIRDEFLRISTASGDGRHYCYPHFTCAVDTENARRIFNDCRDIIQRMHLREY 240

mini-Gs/q58 181 PRVTRAKYFIRDEFLRISTASGDGRHYCYPHFTCAVDTENARRIFAAVKDTILQLNLKEY 240

mini-Gs/q70 181 PRVTRAKYFIRDEFLRISTASGDGRHYCYPHFTCAVDTENARRIFNDCKDIILQMNLREY 240

mini-Gs/q71 181 PRVTRAKYFIRKEFVDISTASGDGRHICYPHFTCAVDTENARRIFNDCKDIILQMNLREY 240

*. ** *. . * . * ***** ****** ** .* * ...*..*

2345678901234567 12345 12345678901234567890123

H4 2 | S6 H5 1 2

2A con ◊◊0

β2 con ◊◊◊

mini-Gq 224 NLV 226

mini-Gs 241 ELL 243

mini-Gs/q57 241 NLV 243

mini-Gs/q58 241 NLV 243

mini-Gs/q70 241 NLV 243

mini-Gs/q71 241 NLV 243

*.

456

H5 2

**S4 Fig.** **Sequence alignment of selected mini-G_s/q_ chimeras used in this study.**
