# Supplementary figures and images for: Mini-G proteins: Novel tools for studying GPCRs in their active conformation
Source: PLoS One. 2017 Apr 20;12(4):e0175642. doi: 10.1371/journal.pone.0175642 (PMC5398546; doi:10.1371/journal.pone.0175642)

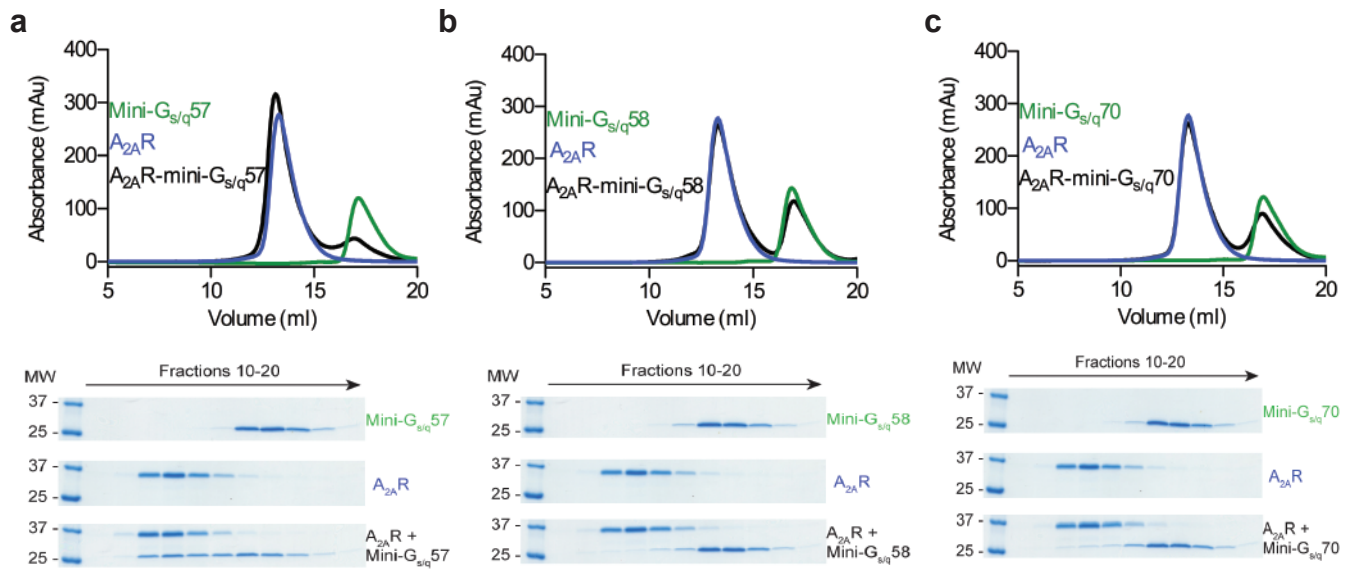

**S5 Fig. Analytical SEC and SDS-PAGE analyses of purified A<sub>2A</sub>R with mini-G<sub>s/q</sub> chimeras.**

Supplement: S5 Fig — Analytical SEC of mini-Gs/q57 (a), mini-Gs/q58 (b) and mini-Gs/q70 (c) bound to purified A2AR: black, A2AR–mini-Gs/q complex; blue, A2AR; green, mini-Gs/q. Three panels below the SEC traces are coomassie blue-stained SDS-PAGE gels of fractions from 3 separate SEC experiments: top panel, mini-Gs/q; middle panel, A2AR; bottom panel, NECA-bound A2AR mixed with mini-Gs/q (1:1.2 molar ratio). (PDF) [file pone.0175642.s005.pdf]

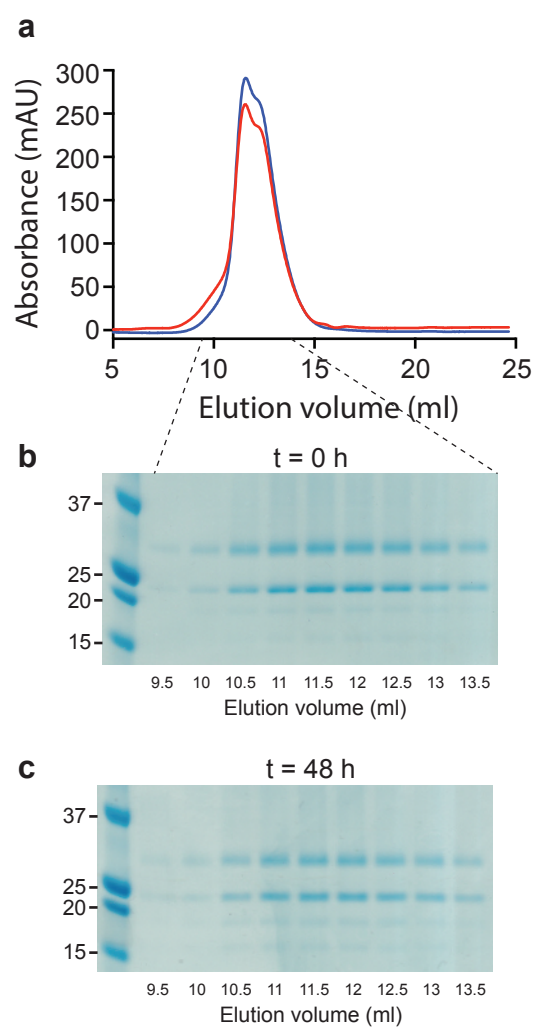

**S7 Fig. Stability of the purified donitriptan-bound 5HT<sub>1B</sub>R-mini-G<sub>01</sub> complex**

Supplement: S7 Fig — (a) The complex was assembled from purified components and analysed by SEC immediately after assembly (blue line) or after the sample was stored at 4°C for 48h (red line). Fractions (0.5 ml) were collected and analysed by SDS-PAGE: (b) immediately after assembly; (c) after 48 h at 4°C. (PDF) [file pone.0175642.s007.pdf]
