# Supplementary material for: Mini-G proteins: Novel tools for studying GPCRs in their active conformation
Source: PLoS One. 2017 Apr 20;12(4):e0175642. doi: 10.1371/journal.pone.0175642 (PMC5398546; doi:10.1371/journal.pone.0175642)
Supplement: S6 Fig — Residues in red are the signature mutations of a mini-G protein. Note the additional G217D mutation (highlighted in yellow; residue 114 in the mini-G protein) in mini-Gi1 to improve expression. Residues highlighted in cyan in mini-Gs were mutated to their equivalent in mini-Gi1 or mini-Go1 (highlighted in magenta and grey respectively) to make the mini-Gs/i1 or mini-Gs/o1 chimeras. Note the re-insertion of the N-terminus and the back mutation (D to L) highlighted in green in the constructs that were used to form a heterotrimer with β1γ2 (i.e. mini-Gi1_46; mini-Gs/i1_43 and mini-Gs/o1_16). (PDF) [file pone.0175642.s006.pdf]

```
miniGs_393      1 MGHHHHHHENLYFQG--------------------IEKQLQKDKQVYRATHRLLLLGADN  40
miniGi1_46      1 MGHHHHHHENLYFQGTLSAED-----KAAVERSKMIDRNLREDGEKAAREVKLLLLGADN  55
miniGs/i1_43    1 MGHHHHHHENLYFQG--------------------IEKQLQKDKQVYRATHRLLLLGADN  40
mniGs/i1_48     1 MGHHHHHHENLYFQGNSKTEDQRNEEKAQREANKKIEKQLQKDKQVYRATHRLLLLGADN  60
miniGo1_12      1 MGHHHHHHENLYFQG--------------------IEKNLKEDGISAAKDVKLLLLGADN  40
miniGs/o_16     1 MGHHHHHHENLYFQGNSKTEDQRNEEKAQREANKKIEKQLQKDKQVYRATHRLLLLGADN  60
                  ***************                    *...*. *        .********
                                 3456789012345678901234567890123   1234567
                               HN2      3         4         5  |   S1    |

miniGs_393     41 SGKSTIVKQMRILHGGSGGSGGTSGIFETKFQVDKVNFHMFDVGGQRDERRKWIQCFNDV 100
miniGi1_46     56 SGKSTIVKQMKIIHGGGGGGGGTTGIVETHFTFKDLHFKMFDVGGQRSERKKWIHCFEDV 115
miniGs/i1_43   41 SGKSTIVKQMRILHGGSGGSGGTSGIFETKFQVDKVNFHMFDVGGQRDERRKWIQCFNDV 100
mniGs/i1_48    61 SGKSTIVKQMRILHGGSGGSGGTSGIFETKFQVDKVNFHMFDVGGQRDERRKWIQCFNDV 120
miniGo1_12     41 SGKSTIVKQMKIIHGGSGGSGGTTGIVETHFTFKNLHFRLFDVGGQRSERKKWIHCFEDV 100
miniGs/o_16    61 SGKSTIVKQMRILHGGSGGSGGTSGIFETKFQVDKVNFHMFDVGGQRDERRKWIQCFNDV 120
                  **********.*.*** ** ***.** **.*    ..*..******* **.***.** **
                    1234567                12345678  12345678   1234567890
                    H1    |                S2     |  S3     |   H2       1

miniGs_393    101 TAIIFVVDSSDYNRLQEALNDFKSIWNNRWLRTISVILFLNKQDLLAEKVLAGKSKIEDY 160
miniGi1_46    116 AAIIFCVDLSDYNRMHESMKLFDSICNNKWFTDTSIILFLNKKDLFEEKIK--KSPLTIC 173
miniGs/i1_43  101 TAIIFVVDSSDYNRLQEALNDFKSIWNNRWLRTISVILFLNKQDLLAEKVLAGKSKIEDY 160
mniGs/i1_48   121 TAIIFVVDSSDYNRLQEALNLFKSIWNNRWLRTISVILFLNKQDLLAEKVLAGKSKIEDY 180
miniGo1_12    101 TAIIFCVDLSDYNRMHESLMDFDSICNNKFFIDTSIILFLNKKDLFGEKIK--KSPLTIC 158
miniGs/o_16   121 TAIIFVVDSSDYNRLQEALNLFKSIWNNRWLRTISVILFLNKQDLLAEKVLAGKSKIEDY 180
                  .**** ** *****..*..  * ** **.     *.******.**  **.   ** .
                   1234567     123456789012345678   1234567 12345678901234567
                  S4     |    H3        1      |   S5    | HG       1      |

miniGs_393    161 FPEFARYTTPEDATPEPGEDPRVTRAKYFIRDEFLRISTASGDGRHYCYPHFTCAVDTEN 220
miniGi1_46    174 YQEYAGSNTYEEAAA-------------YIQCQFEDLNKRKDTKEIYT--HFTCATDTKN 218
miniGs/i1_43  161 FPEFARYTTPEDATPEPGEDPRVTRAKYFIRDEFLRISTASGDGRHYCYPHFTCAVDTEN 220
mniGs/i1_48   181 FPEFARYTTPEDATPEPGEDPRVTRAKYFIRDEFLRISTASGDGRHYCYPHFTCAVDTEN 240
miniGo1_12    159 FPEYTGPNTYEDAAA-------------YIQAQFESKN-RSPNKEIYC--HMTCATDTNN 202
miniGs/o_16   181 FPEFARYTTPEDATPEPGEDPRVTRAKYFIRDEFLRISTASGDGRHYCYPHFTCAVDTEN 240
                  . *..  .* *.*.              .*. .*            *.  * *** ** *
                           123456789012345678901234567          12345     123
                          H4        1         2      |          S6 |    H5

miniGs_393    221 ARRIFNDCRDIIQRMHLRQYELL 243
miniGi1_46    219 AQFIFDAVTDVIIKNNLKDCGLF 241
miniGs/i1_43  221 ARRIFNDVTDIIIKMNLRDCGLF 243
mniGs/i1_48   241 ARRIFNDVTDIIIKMNLRDCGLF 263
miniGo1_12    203 AQVIFDAVTDIIIANNLRGCGLY 225
miniGs/o_16   241 ARRIFNDVTDIIIAMNLRGCGLY 263
                  *. **    *.*   .*.   *
                  45678901234567890123456
                 H5     1         2
```

S6 Fig. Sequence alignment of the different mini-G$_{i1}$ and mini-G$_{o1}$ proteins used in this study.
